# Small Extracellular Vesicles Derived From Damaged Muscle Aggravate Kidney Injury Progression

**DOI:** 10.1002/jcsm.13861

**Published:** 2025-06-13

**Authors:** Songling Jiang, Seunghee Kang, Oran Kwon, Wonhyo Seo, Eun‐Jung Jin, Hunjoo Ha, Joo Young Huh

**Affiliations:** ^1^ Graduate School of Pharmaceutical Sciences, College of Pharmacy Ewha Womans University Seoul Republic of Korea; ^2^ Integrated Omics Institute Wonkwang University Iksan Republic of Korea; ^3^ Department of Nutritional Science and Food Management Ewha Womans University Seoul Republic of Korea; ^4^ Department of Biomedical Materials Science, Graduate School of JABA Wonkwang University Iksan Republic of Korea; ^5^ College of Pharmacy Chung‐Ang University Seoul Republic of Korea; ^6^ Department of Global Innovative Drugs The Graduate School of Chung‐Ang University Seoul Republic of Korea

**Keywords:** extracellular vesicles, kidney injury, microRNA, muscle atrophy, muscle–kidney crosstalk

## Abstract

**Background:**

Muscle atrophy is commonly associated with kidney dysfunction in patients with renal disease. However, the effects of skeletal muscle loss per se on kidney function have not been fully elucidated. Here, we examined muscle–kidney crosstalk by evaluating the role of muscle‐derived small extracellular vesicles (EVs) on the progression of kidney injury.

**Methods:**

A denervation‐induced muscle loss model was established, and kidney inflammation and fibrosis were evaluated in unilateral ureteral obstruction (UUO)‐induced kidney injury and adenine diet‐induced chronic kidney disease models. Changes in small EV markers (CD9, CD63, CD81 and Alix) were measured. GW4869, an inhibitor of EV biogenesis and release, was used to confirm the role of denervated muscle‐derived small EVs on the progression of kidney fibrosis. To obtain direct evidence on the crosstalk, EVs were isolated from plasma of denervated mice and conditioned media from differentiated myotubes and treated in vivo and in vitro. To determine the effect of exercise‐induced EVs on kidney fibrosis, EVs isolated from exercised mice and trained humans were treated to TGFβ1‐stimulated mProx24 renal tubular epithelial cells.

**Results:**

Denervation aggravated kidney injury, as shown by a 10% increase in albuminuria and upregulation of inflammatory and fibrotic markers in the kidney. Significant interactions between denervation and UUO were observed for renal αSMA (*F* = 47.19, *p* < 0.0001) and FN (*F* = 19.06, *p* = 0.0001) expression. EV production and secretion were markedly increased in damaged muscle, both in vivo and in vitro. Pharmacological depletion of EVs using GW4869 via intraperitoneal and intramuscular injection reduced kidney injury by over 20%. Furthermore, injection of EVs from denervated muscle into UUO mice, as well as treatment of mProx24 cells with EVs from either denervated mice or damaged C2C12 myotubes, significantly amplified renal injury. Among the EV cargo, miR‐21a‐3p—identified as a regulator of Ppargc1a—was elevated over 10‐fold in EVs from denervated muscle compared with sham controls. Importantly, EV‐induced injury in mProx24 cells was reversed by pretreatment with a miR‐21a inhibitor. In contrast, exercise downregulated miR‐21a‐3p expression in muscle, and EVs derived from exercised mice and humans attenuated kidney fibrosis.

**Conclusions:**

Our findings provide novel evidence that skeletal muscle loss can serve as an upstream contributor to kidney disease. Muscle‐derived EVs from damaged tissue exacerbate, while those from exercised muscle ameliorate, kidney injury, partly through miR‐21a‐3p regulation. These results highlight the critical role of miRNAs within muscle‐derived EVs in maintaining kidney homeostasis and suggest their potential as therapeutic targets.

## 1 Introduction

Muscle atrophy, characterized by reduced muscle mass and function, is common in kidney disease and linked to higher mortality in dialysis patients [1, 2]. Conversely, exercise therapy improves kidney function and survival in patients [3] and animal models of renal disease [4]. These studies provide evidence for the importance of muscle physiology in regulating kidney homeostasis in patients with renal disease.

Research on muscle–kidney crosstalk has predominantly focused on how kidney dysfunction contributes to muscle atrophy, primarily through circulating uremic toxins, proinflammatory cytokines and reactive oxygen species [5]. A well‐established example is muscle wasting induced by metabolic acidosis in chronic kidney disease (CKD) [6]. In contrast, the potential impact of muscle atrophy itself on kidney function remains largely unexplored.

Skeletal muscle is a major secretory organ that releases proteins/peptides, nucleotides and metabolites, referred to as myokines [7]. These molecules can act locally or on distant tissues, mediating muscle–organ crosstalk [8]. Some of these molecules are contained in small extracellular vesicles (EVs), often referred to as exosomes, from which they are secreted into circulation. EVs are lipid bilayer‐enclosed particles that carry proteins, miRNAs and metabolites and can regulate recipient cell function [9]. Recent studies have shown that exercise and muscle disorders alter the levels of EVs and their contents in muscle. For example, circulating EVs released through high‐intensity interval training in mice, which involved treadmill running 3 days/week for 5 weeks, were shown to carry a specific miRNA signature and induce expression changes in the liver to improve insulin sensitivity [10]. EV production was also increased in the muscle of patients with amyotrophic lateral sclerosis, which disrupted RNA processing, leading to motor neuron cell death [11]. Based on these observations, EV studies can reveal biomarkers reflecting muscular function as well as mediators responsible for interactions between muscle and other organs.

Here, we hypothesized that skeletal muscle injury may serve as an upstream driver of kidney pathology through the release of muscle‐derived small EVs. We constructed a denervation‐induced muscle atrophy model to investigate the impact of muscle loss on kidney injury in both unilateral ureteral obstruction (UUO) and adenine‐induced CKD models. EVs isolated from plasma, denervated muscle and conditioned media of cultured myotubes were used to provide causal evidence for muscle–kidney crosstalk. Finally, miRNA profiling was performed to identify EV cargo, and the role of specific miRNAs in mediating this crosstalk was validated in both denervated and exercised muscle.

## 2 Methods

### 2.1 Animal Experiments

Animal experiments were approved by the Institutional Animal Care and Use Committee of Ewha Womans University (No. 20‐003) and Wonkwang University (WKU23‐61). Male C57BL/6J mice were purchased from Central Lab Animal Inc. (Seoul, Korea) and were acclimated for 1 week. Grip strength was measured using a calibrated grip strength apparatus (BIOSEB, Vitrolles, France). Plasma, kidney and gastrocnemius muscle were collected upon euthanasia. Plasma blood urea nitrogen (BUN) levels were measured using an ELISA kit (Arbor Assays, Ann Arbor, MI, USA).

1 Tibial nerve denervation model

Tibial nerve denervation was performed in 5‐week‐old mice as previously described [12]. Briefly, the right tibial nerve was cut with microdissecting scissors, and the end of the transected tibial nerve was sutured to the adjacent biceps femoris muscle. UUO surgery was performed 7 days after denervation. In a separate study, mice were kept for 4 weeks after denervation, to evaluate the long‐term effect of muscle denervation on kidney.

2 Kidney disease model

UUO surgery was performed as previously described [13], and the mice were euthanized after 5 days. To establish the CKD model, mice were fed a 0.25% adenine‐supplemented diet for 7 days [14].

3 GW4869 administration

GW4869 (Sigma‐Aldrich, St. Louis, MO, USA), an inhibitor of EV biogenesis and release [15], was administered to 5‐week‐old mice at 5 mg/kg/day for 12 days via intraperitoneal or intramuscular injections, starting on the day of denervation. Control mice received equivalent volumes of 0.005% dimethyl sulfoxide (DMSO).

4 Exercise study in mice

Seven‐week‐old mice underwent moderate‐intensity exercise on a RotaRod (Ugo Basile SRL, Gemonio, Italy) at a constant speed of 5.8 m/min for 1 h. For acclimation, mice were placed on the rotating rod at approximately 3 m/min for 5 min 1 day prior. Mice were sacrificed 15 min after completing the exercise [16].

### 2.2 Cell Culture

C2C12 cells (ATCC, Manassas, VA, USA) were maintained at 37°C in a humidified incubator with 5% CO_2_ and cultured in high‐glucose DMEM (Gibco, Waltham, MA, USA) supplemented with 10% fetal bovine serum (Gibco). Myotube differentiation was induced using 2% horse serum for 5 days. For conditioned medium (CM) production, differentiated myotubes were stimulated with dexamethasone or transforming growth factor beta 1 (TGFβ1), washed with filtered phosphate‐buffered saline (PBS) and incubated with EV‐depleted medium for 40 h [17]. EVs were depleted from horse serum by ultracentrifugation at 100 000× *g* for 18 h at 4°C (Type 100 Ti rotor; Beckman Coulter, Indianapolis, IN, USA) [18]. mProx24 mouse renal tubular epithelial cells were supplied by Dr. Takeshi Sugaya (St. Marianna University School of Medicine, Kanagawa, Japan) and cultured in high‐glucose DMEM supplemented with 10% fetal bovine serum [13]. miR‐21a inhibitor (Thermo Fisher Scientific) was treated to C2C12 myotubes or mProx24 cells for 24 h. All in vitro experiments were conducted using mProx cells at passages 23–27 and C2C12 cells at passages 8–10. Each replicate was treated with EVs isolated from individual mouse or human plasma samples.

### 2.3 Human Exercise Studies

The human study adhered to the Declaration of Helsinki and was approved by the Institutional Review Boards of Ewha Womans University Medical Center and registered on the International Clinical Trials Registry Platform (KCT0001137) [19]. Participant characteristics are detailed in Table S1. All subjects provided informed consent. The exercise group performed knee‐strengthening resistance exercises (1 set at 10RM, 3 times/week) and received Korean mistletoe extract (2 g/day) for 12 weeks. The control group received no intervention. Plasma samples were collected for small EV isolation. Also, human muscle data (GSE165632) from the Gene Expression Omnibus were used to analyse miRNA expression among older adult men (aged 65–80 years) with different exercise habits. Specifically, the dataset included five sedentary subjects and nine senior sportsmen, including four predominantly resistance trained and five predominantly endurance trained. Data were analysed using muscle tissues obtained from *vastus lateralis*.

### 2.4 Isolation of Small EVs

Plasma or CM was centrifuged at low speed to remove large debris, filtered through a 0.22 μm filter and purified using commercial kits (EX‐03, EX‐01; Rosetta Exosome Inc., Seongnam, South Korea) or a qEV size exclusion column (qEVsingle/35 nm; IZON, Medford, MA, USA). The column separates particles based on size, retaining those larger than 35 nm. As EVs typically elute early, the first 0.8 mL of eluate was collected. Purified EVs were then resuspended and diluted in filtered PBS. The number of EVs was measured by dynamic light scattering (Zetasizer Nano‐ZS90; Malvern Panalytical Ltd., Malvern, UK) and EV concentration was analysed using a Nanoparticle Tracking Analyser (NS300; Malvern Panalytical Ltd.). To isolate EVs from muscle, freshly harvested gastrocnemius muscle was harvested from mice 2 weeks after denervation and incubated in RPMI 1640 medium (Gibco) containing 2 mg/mL type 2 collagenase (Worthington Biochemical Corporation, Worthington, NJ, USA) and 40 U/mL DNase I (Roche, Basel, Switzerland) at 37°C for 30 min [20]. After differential centrifugation, the supernatants were filtered through a 0.45‐μm membrane and centrifuged at 100 000× *g* for 70 min. Isolated EVs were either used to treat mProx24 cells for 24 h (50 μg) or injected into mice via the tail vein for 5 days (50 μg).

### 2.5 Transmission Electron Microscopy

Isolated EVs were diluted to 1:100 in PBS, and 5 μL diluted EVs were dropped on Formvar–carbon‐coated EM grids. The grids were stained with 2% uranyl acetate for 2 min at RT and removed using filter paper. EV morphology was evaluated by transmission electron microscopy (H‐7650; Hitachi‐Science & Technology, Tokyo, Japan).

### 2.6 Real‐Time PCR (qPCR)

mRNA levels were measured by qPCR using a SYBR Green PCR Master Mix kit (Applied Biosystems, Waltham, MA, USA) in a StepOne real‐time PCR system (Applied Biosystems). mRNA expression was normalized to 18S rRNA levels. We confirmed that 18S rRNA levels did not differ among groups (data not shown). miRNA was extracted from EVs, and cDNA synthesis was conducted using TaqMan Advanced miRNA Assays (Thermo Fisher Scientific, Waltham, MA, USA). miR‐21a‐3p expression was measured by qPCR using a TaqMan Advanced Master Mix 2X (Thermo Fisher Scientific). The primer sequences are listed in Table S2.

### 2.7 Western Blot Analysis

Cell lysate was mixed with 5× sample buffers and heated at 95°C for 6 min. Total protein concentrations were measured using the Bradford method (Bio‐Rad, Hercules, CA, USA), and whole lysates were subjected to SDS‐PAGE. After membrane blocking with 5% skim milk, the target proteins were probed using various antibodies (Table S3). The protein bands were detected using ChemiDoc MP Imaging System (Bio‐Rad). Kidney protein levels were normalized to that of glyceraldehyde 3‐phosphate dehydrogenase (GAPDH).

### 2.8 Immunohistochemistry (IHC) and Immunofluorescence Staining

For IHC staining, anti‐collagen 1 (COL1; SouthernBiotech, Birmingham, AL, USA), anti‐neutrophil gelatinase‐associated lipocalin (NGAL; Abcam, Cambridge, UK) and anti‐alpha‐smooth muscle actin (αSMA; Abcam) antibodies were used. For fluorescence images, anti‐CD63 (Abcam) antibody was used. Sample sections were incubated with primary antibodies overnight and then with Alexa 568‐conjugated anti‐rabbit (Invitrogen, Carlsbad, CA, USA) antibody at room temperature for 1 h. Images were captured using a Zeiss microscope (Carl Zeiss, Oberkochen, Germany). Randomly selected digital images (20–25) from each mouse were quantified using Image‐Pro Plus 4.5 software (Media Cybernetics, Rockville, MD, USA).

### 2.9 Identification of Candidate miRNA

Public RNA‐Seq datasets from the Gene Expression Omnibus database were analysed to identify differentially expressed genes (DEGs) in denervated muscle (GSE62812) and obstructed kidneys (GSE38117). In GSE62812, gastrocnemius muscles were collected 14 days after sciatic nerve denervation or sham surgery (*n* = 3/group), and DEGs were identified by comparing the two groups. Similarly, in GSE38117, gene expression was compared between kidneys with and without UUO (*n* = 3/group). miRNA expression changes were analysed in denervated muscle (GSE162794, *n* = 4/group) and obstructed kidneys (GSE42719, *n* = 4/group). Differential expression was considered significant with a fold change ≥ 1.5 or ≤ 0.8 and adjusted *p*‐value < 0.05. Predicted miRNAs targeting *Ppargc1a* were validated using miRbase (https://www.mirbase.org/). Gene ontology enrichment analysis of 155 candidate genes was performed using g: Profiler (https://biit.cs.ut.ee/gprofiler/gost) [21].

### 2.10 Statistical Analysis

All results are expressed as the mean ± standard error (SE). Differences among multiple groups were assessed using one‐way or two‐way analysis of variance (ANOVA) in GraphPad Prism (version 8.0.2), followed by Tukey's post hoc test for pairwise comparisons. A two‐tailed Pearson correlation analysis was used to assess the relationship between biomarkers. Statistical significance was set at *p* < 0.05.

## 3 Results

### 3.1 Tibial Nerve Denervation Leads to Muscle Atrophy in Control and UUO Mice

**FIGURE 1:**
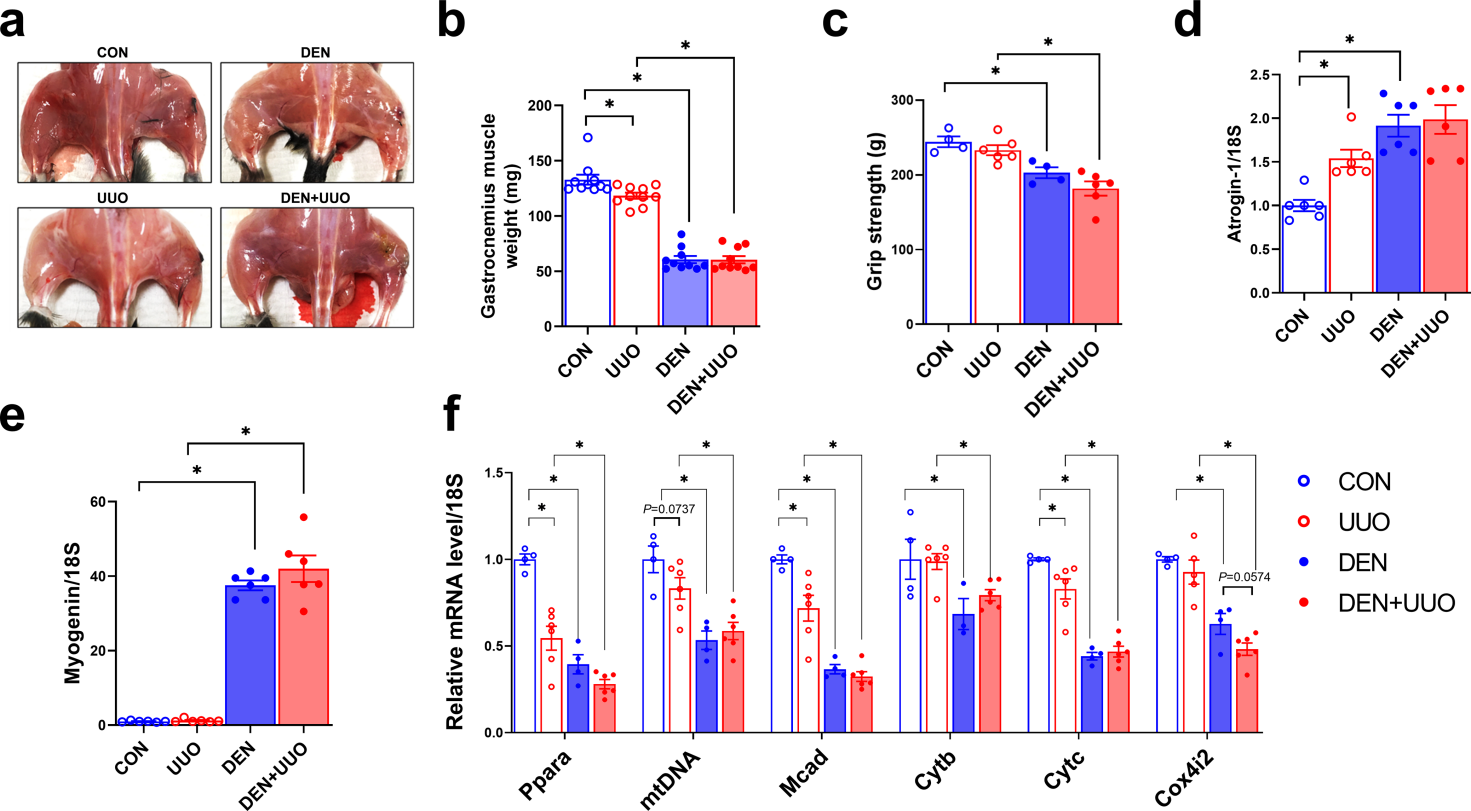
Tibial nerve denervation leads to muscle atrophy. (a) Scheme of experimental design. UUO was conducted 7 days after muscle denervation, and mice were euthanized 5 days after UUO surgery. (b) Gastrocnemius muscle weight and (c) strength were measured in mice after 5 days of UUO. (d, e) *Atrogin‐1* and *Myogenin* expressions were measured using qPCR. (f) Mitochondria‐related genes were measured and normalized to their respective 18S rRNA. Data are expressed as the mean ± standard error of 5–6 mice/group. Statistical significance was determined using two‐way ANOVA. *p < 0.05. The experimental groups were defined as follows: CON (Control), UUO (Unilateral Ureteral Obstruction only), DEN (Muscle denervation only) and DEN + UUO (UUO surgery performed after muscle denervation).

To determine the effect of muscle wasting on kidney injury progression, we performed UUO in tibial nerve‐denervated mice (Figure 1a). Muscle atrophy was confirmed by lower muscle mass and strength in both DEN and DEN + UUO groups compared with control (Figure 1b,c). *Atrogin‐1* and *Myog* expressions were higher (Figure 1d,e), whereas mitochondria‐related gene expressions were lower in denervated mice (Figure 1f). Two‐way ANOVA revealed significant interactions between denervation and UUO for *Ppara* (*F* = 10.55, *p* = 0.005), *Mcad* (*F* = 5.349, *p* = 0.0344) and *Cytc* (*F* = 5.467, *p* = 0.0327). UUO alone caused mild but significant muscle injury, evidenced by lower muscle weight and higher *Atrogin‐1* expression (Figure 1b,d), along with lower expression of *Ppara*, *Mcad* and *Cytc* (Figure 1f). However, muscle strength in UUO mice without denervation did not differ from control (Figure 1c). Notably, the extent of muscle injury was comparable between DEN and DEN + UUO groups.

### 3.2 Muscle Denervation Aggravates Kidney Injury

**FIGURE 2:**
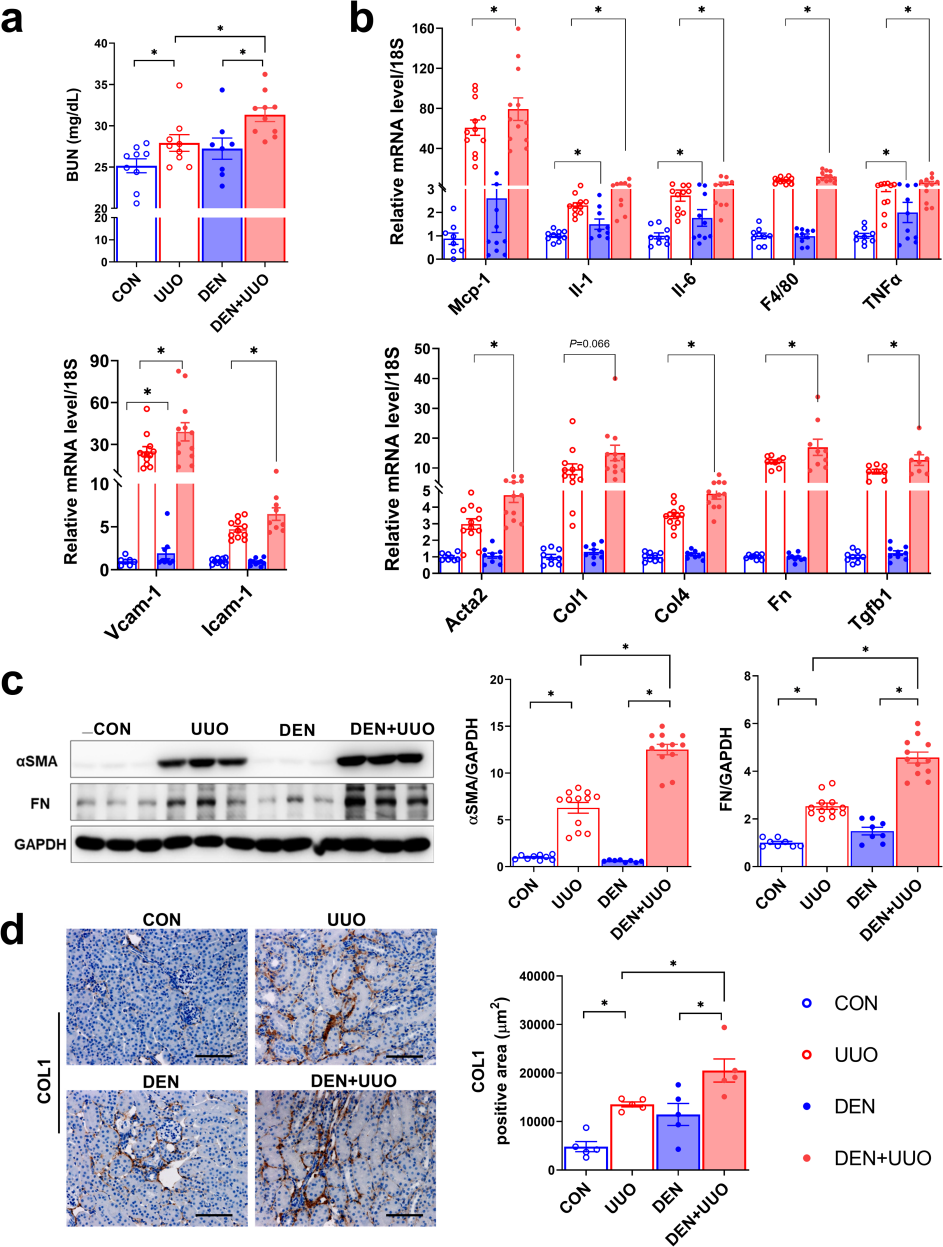
Reduced muscle mass aggravates UUO‐induced kidney injury. (a) Plasma BUN was measured using ELISA. (b) Expressions of genes related to kidney inflammation and fibrosis were measured using qPCR. (c) αSMA and FN protein levels were measured using western blotting, and the results were normalized to GAPDH. Data are expressed as the mean ± standard error of 10–12 mice/group. (d) Kidney sections were stained with anti‐COL1 and quantified. Original magnification, 100×; scale bar, 200 μm. Data are expressed as the mean ± standard error of five mice/group. Statistical significance was determined using two‐way ANOVA. *p < 0.05. The experimental groups were defined as follows: CON (Control), UUO (Unilateral Ureteral Obstruction only), DEN (Muscle denervation only) and DEN + UUO (UUO surgery performed after muscle denervation). αSMA, alpha‐smooth muscle actin; BUN, blood urea nitrogen; FN, fibronectin; GAPDH, glyceraldehyde‐3‐phosphate dehydrogenase.

Next, we examined the effect of muscle denervation on the kidney. Elevated plasma BUN levels in UUO mice were aggravated in DEN + UUO mice (Figure 2a). UUO‐induced kidney inflammation, as indicated by upregulated *Mcp‐1*, *Il‐1*, *Il‐6*, *F4/80*, *Tnfa*, *Vcam‐1* and *Icam‐1*, and fibrosis markers, including *Acta2*, *Col1*, *Col4*, *Fn* and *Tgfb1*, were higher in DEN + UUO mice compared with UUO alone (Figure 2b). Comparable results were observed for αSMA, fibronectin (FN) and COL1 protein levels (Figure 2c,d), with significant interactions between denervation and UUO for αSMA (*F* = 47.19, *p* < 0.0001) and FN (*F* = 19.06, *p* = 0.0001). Similar findings were observed in the adenine diet‐induced CKD model, where denervation further elevated BUN levels and enhanced expression of inflammation and fibrosis markers in the kidneys of adenine‐fed mice (Figure S1).

Notably, 12 days of denervation alone was sufficient to induce *IL‐1*, *IL‐6*, *TNFα* and *Vcam‐1* expression in the kidney, along with higher protein levels of αSMA, FN and COL1 (Figure 2b–d). To determine whether prolonged denervation could further exacerbate renal injury, a separate cohort of mice was observed for 4 weeks post‐denervation without UUO surgery or adenine‐diet supplement (Figure S2). Compared with control, denervated mice exhibited significantly lower body weight and muscle strength (Figure S2a,b), along with increased albuminuria (Figure S2c). Consistently, renal expression of inflammatory and fibrotic genes was elevated in these mice (Figure S2d). These findings support a causative role of denervated muscle in the development of kidney injury.

### 3.3 Production and Release of Small EVs Is Increased in Damaged Muscle

**FIGURE 3:**
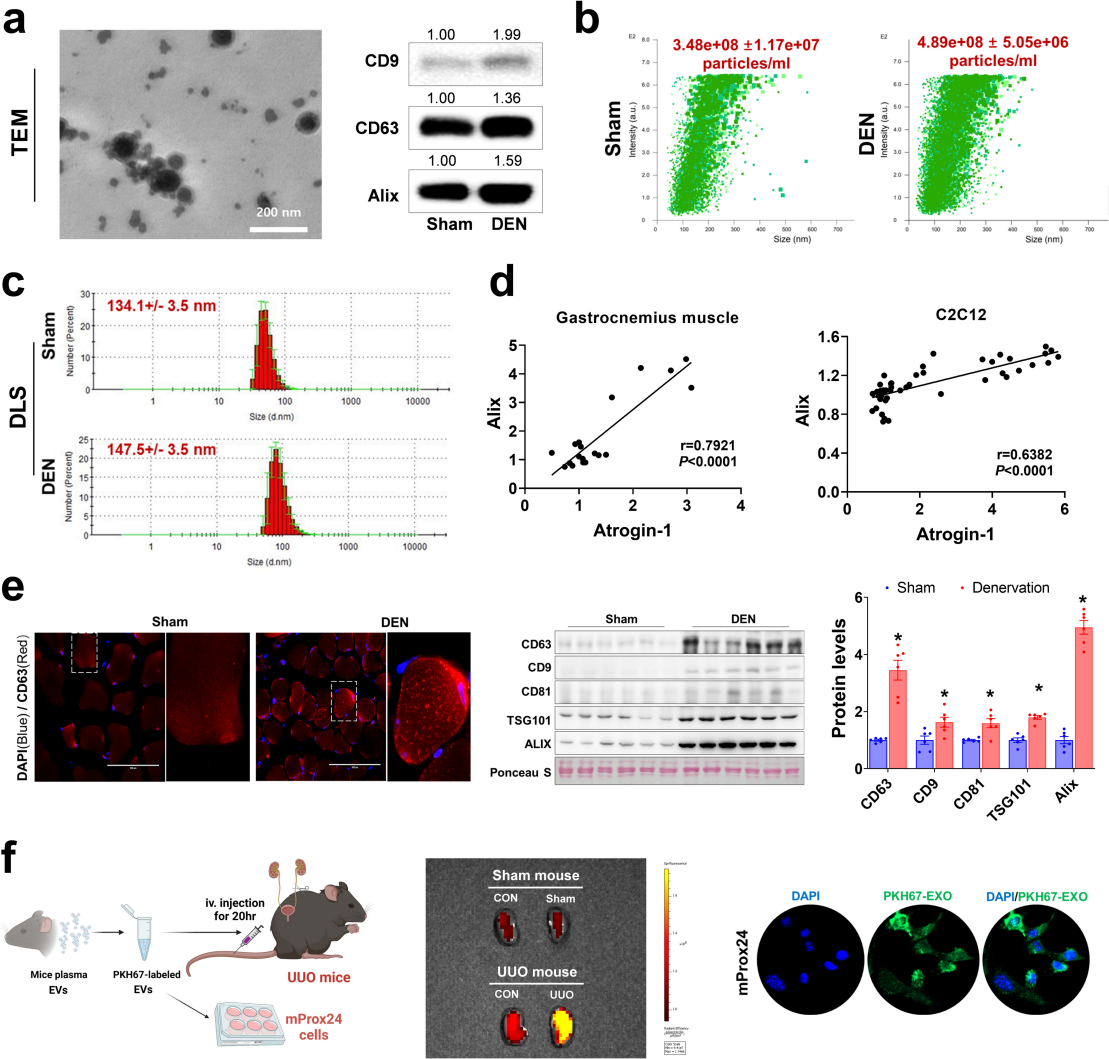
Muscle denervation leads to increased production and release of small EVs. (a) Isolated plasma EV morphology was visualized with TEM. Circulating small EV marker protein levels were measured by western blotting. (b) Number of plasma EVs from sham and denervated mice was determined using NanoSight. (c) DLS analysis showing the number distributions of plasma EVs. (d) Scatter plot showing a positive correlation between *Alix* and *Atrogin‐1* in gastrocnemius muscle (*n* = 20) and myotubes (*n* = 45). Gene expressions were measured using qPCR. A two‐tailed Pearson correlation analysis was used to assess the relationship between Alix and Atrogin‐1. (e) Representative immunofluorescence images of CD63 in sham and denervated muscle. Small EV marker protein levels in gastrocnemius muscle were measured by western blotting. Ponceau red staining showed that equivalent amounts of protein were loaded (*n* = 6). (f) Experimental design. Mouse plasma EVs were isolated and labelled with PKH67. Fluorescence image of sham and UUO kidneys acquired 20 h post‐intravenous injection of PKH67‐labelled EVs. Fluorescence image of mProx24 cells after 1 h treatment with PKH67‐labelled EVs. Cell nuclei were incubated with DAPI (2 μg/mL, 2 min). Data are expressed as the mean ± standard error. Statistical significance was determined using one‐way ANOVA followed by Tukey's post hoc test for multiple comparisons. *p < 0.05 vs. sham. EVs, extracellular vesicles; DEN, denervation; TEM, transmission electron microscopy; UUO, unilateral ureteral obstruction.

We hypothesized that small EVs from denervated muscle could mediate crosstalk with the kidney. To test this hypothesis, we first isolated EVs from mouse plasma. The expression of small EV markers (CD9, CD63, CD81 and Alix), as well as EV particle number, was higher in plasma EVs isolated from denervated mice compared with sham controls (Figure 3a,b), while average vesicle size was unaltered (Figure 3c). Notably, *Alix* and *Atrogin‐1* expression levels were positively correlated in both gastrocnemius muscle (*r* = 0.7921, *p* < 0.0001) and differentiated myotubes (*r* = 0.6382, *p* < 0.0001) (Figure 3d). Consistently, muscle protein EV markers were higher in denervated mice compared with sham controls (Figure 3e). Gene expression also confirmed that muscle EV production is induced by denervation and unaffected by UUO (Figure S3a). In contrast, kidney EV production was significantly induced by UUO (Figure S4). Next, we treated cultured myotubes with dexamethasone, a synthetic glucocorticoid known to induce muscle injury [22], or TGFβ1, which cause both muscle wasting [23] and kidney fibrosis [13]. As expected, these treatments induced the expression of EV markers (Figure S3b,c). Notably, genes related to EV secretion (*Rab11a*, *Rab11b* and *Rab35*) were also increased.

We further examined whether small EVs could be targeted to the injured kidney. PKH67‐labelled plasma EVs from control mice were intravenously administrated to UUO mice (Figure 3f). EVs accumulated in the obstructed kidney but not in the control kidney, as indicated by higher fluorescence intensity (1.57 × 10^8^ vs. 1.03 × 10^8^). mProx24 proximal tubular epithelial cells were also treated with plasma EVs for 1 h, confirming EV uptake into the cells (Figure 3f). Collectively, these results imply that EVs could be effectively delivered to the damaged kidney.

### 3.4 Inhibition of Small EV Generation and Release Ameliorates Kidney Injury in Denervated UUO Mice

**FIGURE 4:**
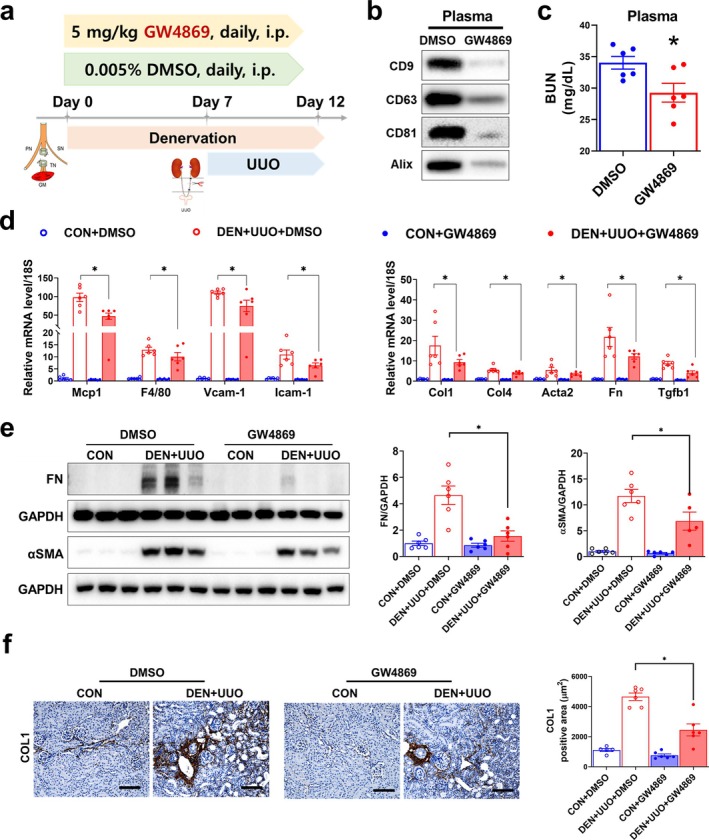
Inhibition of EV synthesis/secretion by GW4869 inhibits kidney inflammation and fibrosis in denervated UUO mice. (a) Scheme of experimental design. Denervated UUO mice were subjected to daily intraperitoneal injections of GW4869 or DMSO. (b) Small EV markers were measured by western blotting in the plasma EVs of DMSO‐ and GW4869‐treated mice. (c) Plasma BUN was measured using ELISA. (d) Expressions of genes related to kidney inflammation and fibrosis were measured using qPCR. (e) FN and αSMA protein levels were measured by western blotting, and the results were normalized to GAPDH. (f) Kidney sections were stained with anti‐COL1 and quantified. Original magnification, 100×; scale bar, 200 μm. Data are expressed as the mean ± standard error of six mice/group. Statistical significance was determined using one‐way ANOVA followed by Tukey's post hoc test for multiple comparisons. *p < 0.05. BUN, blood urea nitrogen; EVs, extracellular vesicles; UUO, unilateral ureteral obstruction.

Although muscle injury promotes EV production and release, it remains unclear whether these EVs contribute to the progression of renal injury. To address this, we utilized GW4869 to block EV generation and release [15] (Figure 4a). Circulating EVs were effectively removed by intraperitoneal GW4869 administration, confirmed by diminished EV marker expression in plasma (Figure 4b). Plasma BUN levels were lower in GW4869‐treated denervated UUO mice compared with DMSO‐treated controls (Figure 4c). Genetic markers of inflammation and fibrosis were significantly lower in GW4869‐treated mice (Figure 4d). Partial inhibition of induced genes implied the involvement of non‐EV factors in muscle–kidney crosstalk. The anti‐fibrotic effect of GW4869 was more evident in protein levels, where the elevated expression of FN, αSMA and COL1 in denervated UUO mice was attenuated by GW4869 treatment (Figure 4e,f). As plasma EVs could be derived from tissues other than muscle, we repeated the experiments by intramuscularly injecting GW4869. Diminished CD63 and TSG101 expression in muscle and plasma, respectively, confirmed that the EVs were effectively removed (Figure S5a,b). Similar to the intraperitoneal injection results, upregulated inflammatory and fibrotic genes in denervated UUO mice kidneys were significantly reduced by EV depletion in muscle (Figure S5c). Moreover, the Masson's trichrome and αSMA stained areas induced by denervation in UUO mice were completely ameliorated (Figure S5d,e). These results indicate that plasma EVs in denervated mice are mostly derived from muscle, and their depletion can attenuate the effect of muscle denervation on renal injury.

### 3.5 Treatment With EVs Isolated From Damaged Muscle Aggravates Fibrosis in Cultured Proximal Tubular Epithelial Cells and Mouse Kidneys

**FIGURE 5:**
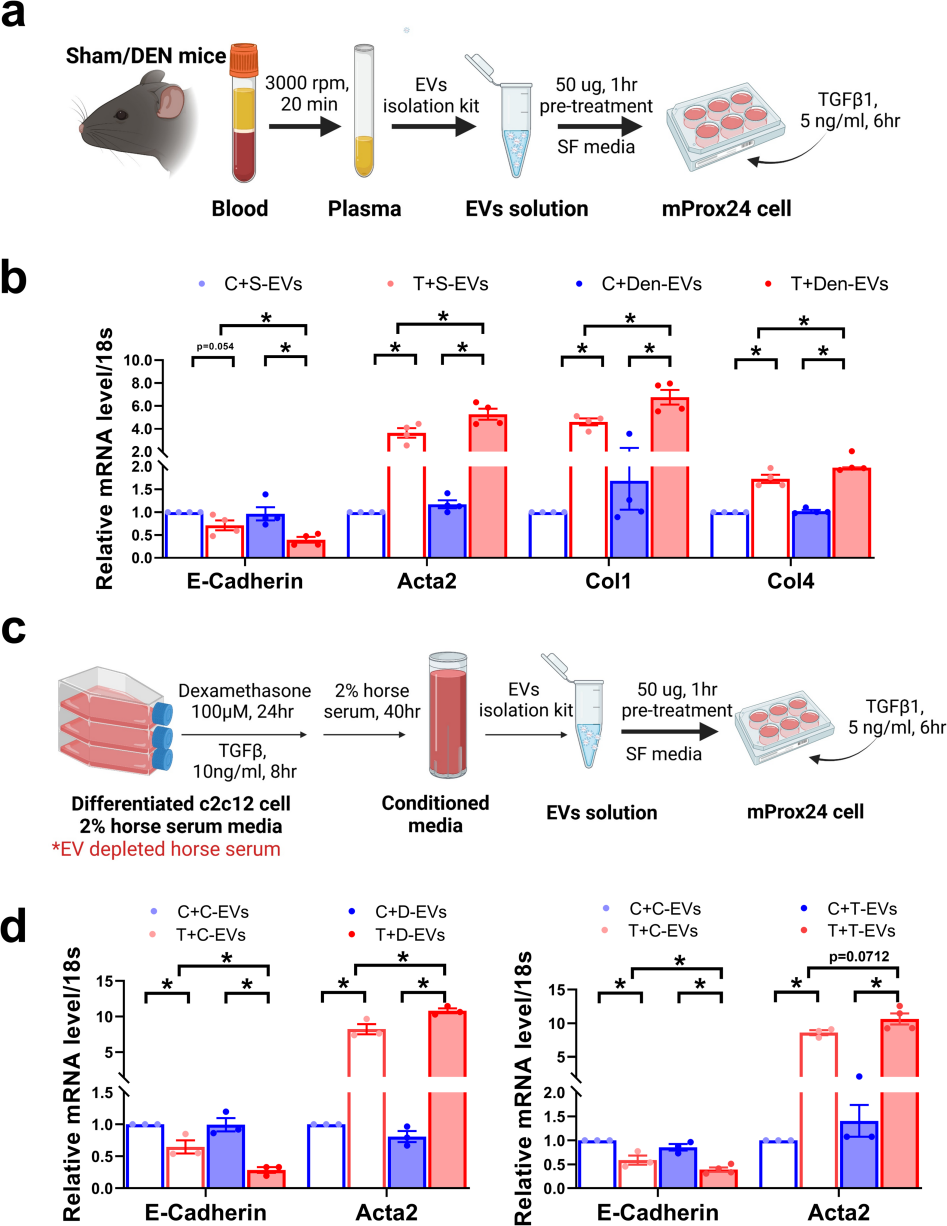
Small EVs from damaged muscle aggravate fibrosis in mProx24 cells. (a) mProx24 cells were pretreated with 50 μg of plasma EVs from sham or denervated mice for 1 h and then stimulated with TGFβ1 (5 ng/mL, 6 h). (b) *E‐cadherin*, *Acta2*, *Col1* and *Col4* levels were measured using qPCR and normalized to their respective 18S rRNAs. Data is expressed as the mean ± standard error of four sets. (c) Differentiated myotubes were treated with dexamethasone (100 μM) or TGFβ1 (10 ng/mL) for 24 h, after which the EVs were isolated from conditioned medium for the treatment of mProx24 cells. (d) *E‐cadherin* and *Acta2* levels were measured using qPCR. Data are expressed as the mean ± standard error of three sets. Statistical significance was determined using one‐way ANOVA followed by Tukey's post hoc test for multiple comparisons. *p < 0.05. C, control; D, dexamethasone; EVs, extracellular vesicles; T, TGFβ1.

**FIGURE 6:**
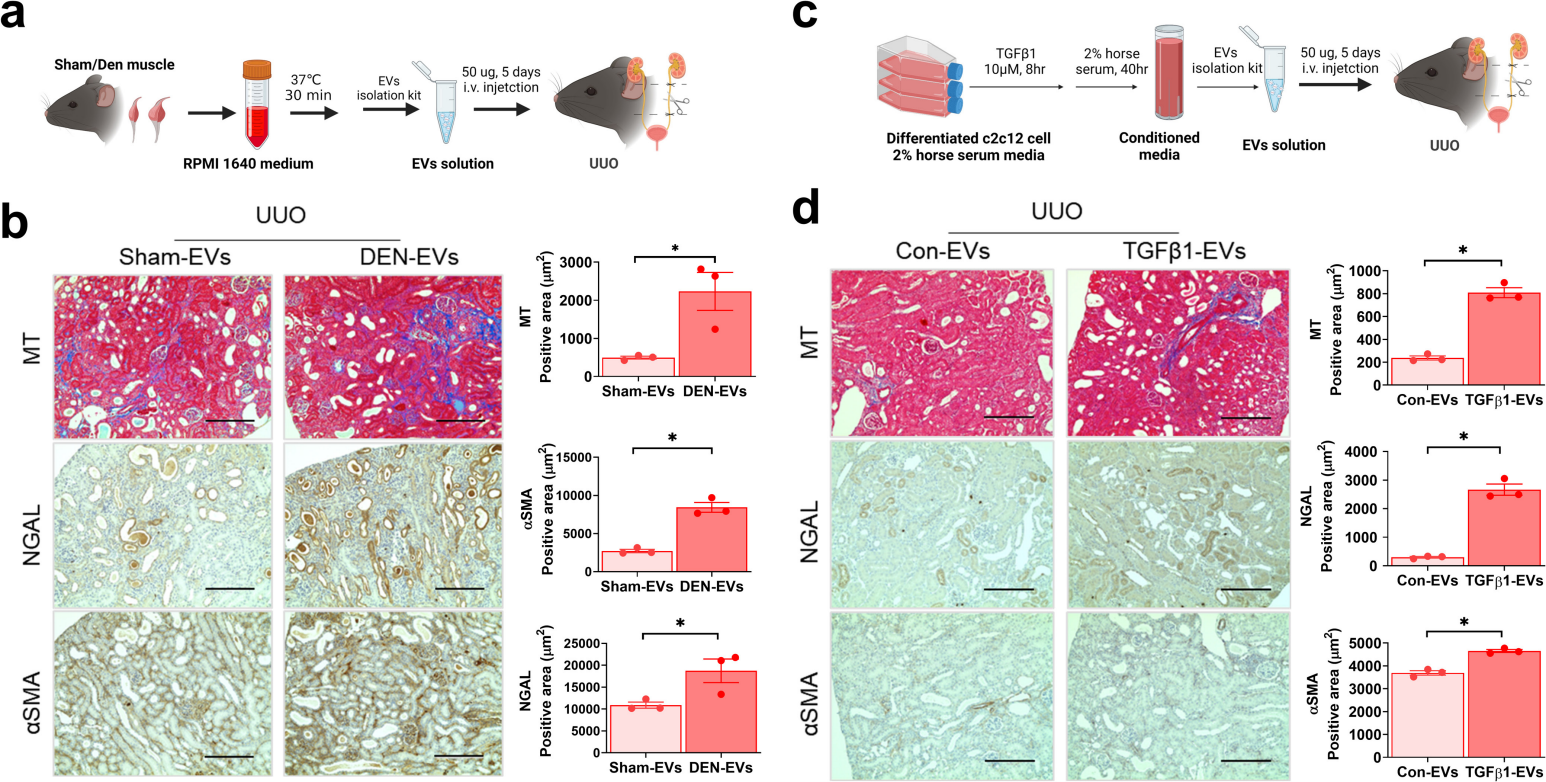
Small EVs from damaged muscle aggravate fibrosis in mouse kidney. (a, b) EVs from gastrocnemius muscle of sham or denervated mice were i.v injected into UUO mice for 5 days. Kidney sections were stained with Masson's trichrome, anti‐NGAL and anti‐αSMA antibody. (c, d) EVs isolated from the conditioned medium of control or TGFβ1‐treated C2C12 myotubes were injected to UUO mice for 5 days. Kidney sections were stained with Masson's trichrome, anti‐NGAL and anti‐αSMA antibody. Data are expressed as the mean ± standard error of three sets. Statistical significance was determined using one‐way ANOVA followed by Tukey's post hoc test for multiple comparisons. *p < 0.05. αSMA, alpha‐smooth muscle actin; EVs, extracellular vesicles; MT, Masson's trichrome; NGAL, neutrophil gelatinase‐associated lipocalin.

To provide direct evidence for the role of EVs in muscle–kidney crosstalk, mProx24 cells were used (Figure 5a). The TGFβ1‐induced downregulation of *E‐cadherin* and upregulation of *Acta2*, *Col1* and *Col4* in mProx24 cells were aggravated following pretreatment with plasma EVs from denervated mice compared with EVs from sham controls (Figure 5b). Next, we isolated EVs from the CM of C2C12 myotubes (Figure 5c). EVs isolated from the CM of dexamethasone‐ and TGFβ1‐treated myotubes aggravated *E‐cadherin* downregulation and *Acta2* upregulation in TGFβ1‐treated mProx24 cells (Figure 5d). The pathogenic role of muscle‐derived EVs was evaluated in vivo by injecting the EVs isolated from denervated muscle (Figure 6a) and TGFβ‐treated C2C12 myotubes (Figure 6c). Five‐day injection of damaged muscle‐derived EVs resulted in aggravated UUO‐induced expression, as shown by Masson's trichrome, NGAL and αSMA staining, compared with EVs from sham mice (Figure 6b) or control myotubes (Figure 6d).

### 3.6 Increased miR‐21a‐3p in EVs From Damaged Muscle Accelerates Kidney Injury

**FIGURE 7:**
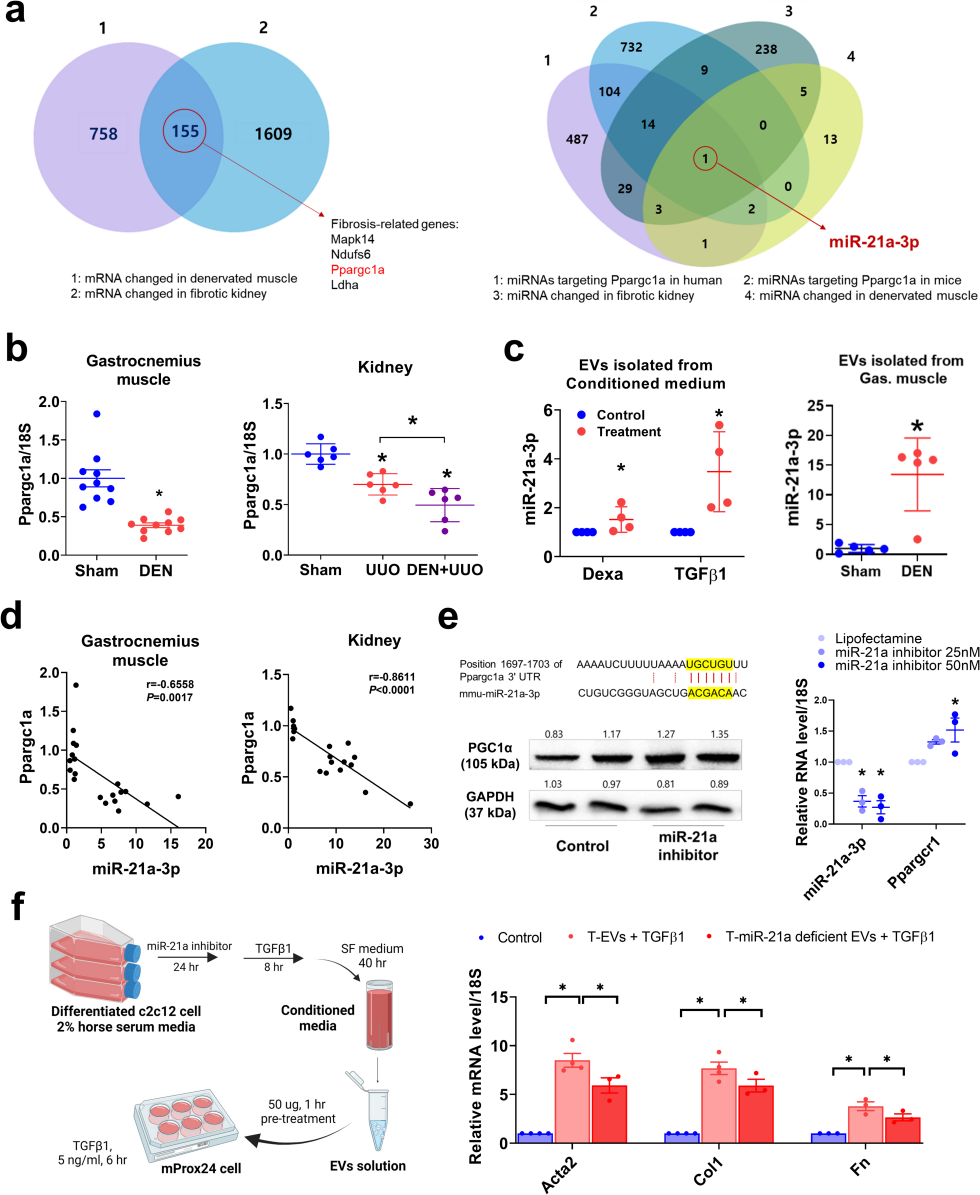
miR‐21a aggravates kidney injury progression through EV‐mediated muscle–kidney crosstalk. (a) Venn diagram showing commonly downregulated genes in denervated muscle and fibrotic kidney, including *Ppargc1a*. Venn diagram showing commonly upregulated miRNAs in denervated muscle and fibrotic kidney, and miRNAs targeting Ppargc1a in humans and mice. (b) *Ppargc1a* expression in denervated muscle (*n* = 10) and obstructed kidney (*n* = 6) measured using qPCR. (c) miR‐21a‐3p expression was measured in EVs isolated from the conditioned medium of damaged myotubes and denervated muscle using qPCR. (d) Scatter plot showing a positive correlation between *Ppargc1a* and miR‐21a‐3p in denervated muscle and obstructed kidney. A two‐tailed Pearson correlation analysis was used to assess the relationship between *Ppargc1a* and miR‐21a‐3p. (e) Predicted binding sites of miR‐21a‐3p on the 3′‐UTR of *Ppargc1a* mRNA, and PGC1α protein levels in mProx24 cells treated with or without miR‐21a inhibitor measured by western blotting. miR‐21a‐3p and *Ppargc1a* expression were measured in myotubes treated with or without miR‐21a inhibitor using qPCR. (f) In vitro experimental design and the expression of fibrotic genes in TGFβ‐induced mProx24 cells treated with EVs enriched or deficient in miR‐21a. Data are expressed as the mean ± standard error of three sets. Statistical significance was determined using one‐way ANOVA followed by Tukey's post hoc test for multiple comparisons. *p < 0.05. EVs, extracellular vesicles; T, TGFβ1.

We hypothesized that the increased miRNAs within EVs could mediate the muscle–kidney crosstalk. As the increased miRNAs would decrease target mRNAs in both muscle and kidney, we first identified the genes commonly downregulated in denervated muscle and fibrotic kidney (Figure 7a and Table S4). Functional enrichment analysis showed that the 155 commonly downregulated genes were enriched in mitochondrial function (Figure S6). Thus, we narrowed down the gene list to those related to mitochondria and fibrosis. Among the four genes related to fibrosis (*Mapk14*, *Ndufs6*, *Ppargc1a* and *Ldha*), *Ppargc1a* was the only one associated with kidney fibrosis [24, 25]. Among the miRNAs commonly upregulated in denervated muscle and kidney fibrosis (Table S5), we screened for those targeting *Ppargc1a* and identified miR‐21a‐3p. To confirm, we measured *Ppargc1a* expression in denervated muscle and obstructed kidney, both of which exhibited significantly lower levels compared with sham controls (Figure 7b). miR‐21a‐3p content was higher in EVs isolated from the CM of dexamethasone‐ and TGFβ1‐treated C2C12 myotubes, as well as in EVs from denervated muscle, compared with controls or sham mice (Figure 7c). A negative correlation was observed between *Ppargc1a* and miR‐21a‐3p expression in both muscle (*r* = −0.6558, *p* = 0.0017) and kidney (*r* = −0.8611, *p* < 0.0001) (Figure 7d). Treatment with miR‐21a inhibitor resulted in increased PGC1α protein in mProx24 cells and *Ppargc1a* gene expression in myotubes (Figure 7e). These results show that miR‐21a‐3p, as cargo in small EVs, is upregulated in damaged muscle and targets *Ppargc1a* to downregulate its expression.

Next, two strategies were used to examine the role of miR‐21a‐3p‐containing EVs in mediating kidney injury progression. First, C2C12 myotubes were treated with miR‐21a inhibitor before isolating EVs from CM (Figure 7f). Second, mProx24 cells were pretreated with miR‐21a inhibitor before stimulation with EVs isolated from either denervated mice or the CM of C2C12 myotubes (Figure S7a). In both cases, TGFβ1‐induced mProx24 injury was ameliorated by depleting miR‐21a (Figure 7f and Figure S7b–d), implying that increased miR‐21a‐3p is the major component of muscle‐derived EVs responsible for kidney fibrosis progression.

### 3.7 EVs Derived From Exercised Mice and Humans Ameliorate TGFβ1‐Induced Kidney Cell Injury

**FIGURE 8:**
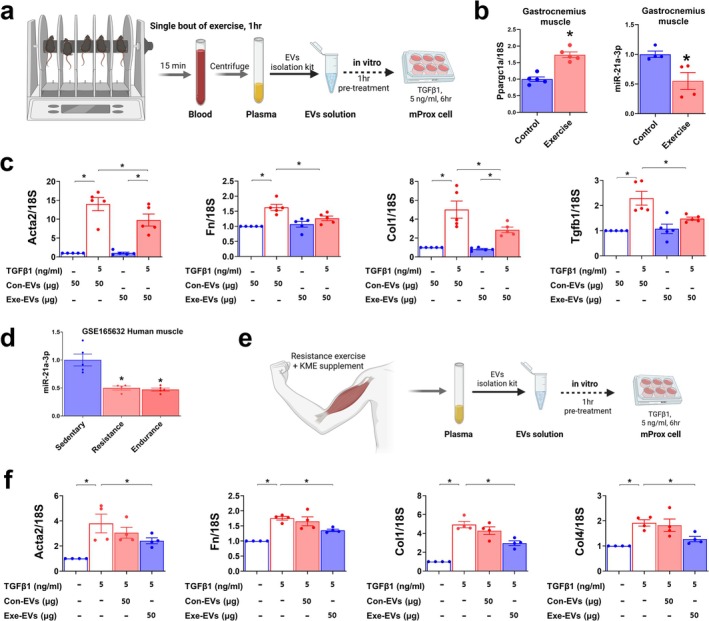
Plasma EVs derived from exercise mice and humans ameliorate TGFβ1‐induced fibrosis in mProx24 cells. (a) Schematic experimental design. Exercise‐induced EVs were purified from mouse plasma and used to pretreat mProx24 cells before stimulation with TGFβ1. (b) *Ppargc1a* and miR‐21a‐3p expression was measured in gastrocnemius muscle using qPCR. (c) mProx24 cells were pretreated with isolated plasma EVs from control (Con‐EVs) and exercised (Exe‐EVs) mice. Next, TGFβ1‐induced expression of *Acta2*, *Fn*, *Col1* and *Tgfb1* was measured using qPCR. Data is expressed as the mean ± standard error of five sets. *p < 0.05. (d) miR‐21a‐3p expression in the muscle of sedentary subjects and humans subjected to resistance and endurance exercises (GSE165632). (e) Schematic experimental design. Exercise‐induced EVs were purified from human plasma and used to pretreat to mProx24 cells before stimulation with TGFβ1. (f) mProx24 cells were pretreated with isolated plasma EVs from control (Con‐EVs) and exercised (Exe‐EVs) humans. Next, TGFβ1‐induced expression *Acta2*, *Fn*, *Col1* and *Col4* was measured using qPCR. Data are expressed as the mean ± standard error of four sets. Statistical significance was determined using one‐way ANOVA followed by Tukey's post hoc test for multiple comparisons. *p < 0.05.

While muscle atrophy can aggravate kidney disease, exercise has beneficial effects in ameliorating kidney disease [26]. Therefore, we sought to determine the effect of exercise‐induced EVs on kidney fibrosis (Figure 8a). In mice, a single bout of RotaRod exercise effectively upregulated *Ppargc1a* and reduced miR‐21a‐3p levels in muscle (Figure 8b). Next, mProx24 cells were treated with plasma EVs isolated from these mice. TGFβ1‐induced *Acta2*, *Fn*, *Col1* and *Tgfb1* expression was inhibited by EVs from exercised mice compared with those from control mice (Figure 8c). Consistent with the mouse data, data mining revealed that muscle miR‐21a‐3p expression was lower in senior sportsmen trained primarily in either resistance or endurance exercise compared with sedentary subjects (Figure 8d). We previously reported that resistance exercise combined with Korean mistletoe extract (KME) supplementation mitigates age‐related muscle loss and strength decline [19]. We utilized the plasma from these individuals to isolated EVs (Figure 8e). Compared with controls, higher muscle mass and lower fat mass was observed in the exercised group (Table S1) Treatment of mProx24 cells with isolated EVs resulted in lower expression of TGFβ1‐induced genes when using EVs from the exercise plus KME group compared with those from the controls (Figure 8f). These results partly show that miR‐21a‐3p inhibition and small EV content regulation in muscle could account for the anti‐fibrotic effect of exercise on the kidney.

## 4 Discussion

In this study, we demonstrated that muscle denervation not only induces local atrophy but also exacerbates renal damage. Muscle denervation exacerbated renal inflammation and fibrosis in both UUO and adenine‐induced CKD models. Our data indicate that EVs from damaged muscle exacerbate kidney injury, with miR‐21a‐3p as a cargo responsible for the crosstalk. Conversely, EVs from exercised muscle alleviated kidney fibrosis, partly through reduced miR‐21a‐3p levels. Our findings highlight a previously underrecognized role of skeletal muscle in modulating kidney injury, demonstrating that in the context of muscle wasting or denervation, skeletal muscle may be a key initiator of renal damage.

While the association between muscle atrophy and CKD is well documented, studies investigating the direct impact of muscle‐derived factors on kidney function remain limited. Myokine irisin ameliorates kidney injury progression by suppressing metabolic reprogramming and fibrogenesis [13, 27]. Meanwhile, muscle‐specific Akt1 overexpression in mice showed that muscle growth per se can attenuate renal injury [28]. Specifically, activation of Akt1 upregulates serum stromal cell‐derived factor‐1, IL‐10 and IL‐17 levels, but whether these factors are directly involved in muscle–kidney crosstalk is unknown. Here, we adopted a denervation model to initiate the damage response in muscle and distinguish the effect of muscle atrophy on the kidney. Albuminuria and markers of inflammation and fibrosis markers were significantly induced in the kidney after 4 weeks of denervation (Figure S2), implying that muscle denervation alone can induce kidney damage through secretory factors from muscle.

Studies have shown that circulating EVs are altered in muscle disorders [7]. Here, we observed elevated plasma and muscle EV levels in denervated mice and confirmed the pathogenic role of muscle‐derived EVs by directly injecting damaged muscle‐derived EVs into UUO mice in vivo and treating TGFβ1‐induced mProx24 cells with isolated EVs in vitro. The migration of EVs at injured sites, confirmed by PKH67‐labelled EVs, may explain why EV increase following denervation in the sham group did not induce kidney injury but did in the UUO or adenine‐fed group. Notably, the autocrine/paracrine effect of kidney‐derived EVs in the regulation of kidney function has been previously described [29, 30], and we also observed increased EV markers in the kidney of UUO mice. The fact that the source of small EVs in plasma cannot be distinguished, whether from muscle, kidney, or other tissues, complicates explaining the effect of denervation on crosstalk. However, EV markers in muscle were higher in denervated mice but not UUO mice, and depletion of circulating and muscle EVs by GW4869 injection resulted in similar levels of inhibition in kidney injury, corroborating EV secretion by denervated muscle as largely accounting for muscle–kidney crosstalk.

Pathological events, including injury, atrophy and aging, can alter the cargo of muscle‐derived EVs [31]. However, the mechanistic aspect of miRNAs as cargo in EVs from atrophic muscles has not been fully explained. Here, we identified miR‐21‐3p as potential cargo, and its role in muscle–kidney crosstalk was confirmed by the depletion of miR‐21‐3p from EVs derived from damaged myotubes. miR‐21 is an established profibrotic miRNA [32], and its inhibition in muscle satellite cells from elderly mice reportedly exerts beneficial effects on myogenesis [33]. We also showed that exercise lowers the expression of miR‐21a‐3p levels in muscle, and plasma EVs derived from exercised subjects ameliorate kidney fibrosis, complementing the mechanisms involved in the beneficial effects of exercise [26]. miR‐21 expression is induced in Duchenne muscular dystrophy and controls age‐associated muscle fibrosis and dystrophy progression [34], while studies have also reported its involvement in kidney fibrosis [35]. Chau et al. identified *Pparα* and *Mpv* as key miR‐21 targets involved in regulating lipid metabolism and mitochondrial reactive oxygen species generation. Here, miR‐21‐3p was selected as a target by screening for *Ppargc1a*, which is also closely associated with lipid metabolism and mitochondria function in the kidneys [25]. miRNA biogenesis produces a guide (sense, miR‐#‐3p) and a passenger (antisense, miR‐#‐5p) strand. While the passenger strand was traditionally thought to be degraded, recent research showed it can also accumulate and regulate target mRNAs [36], sometimes exerting effects opposite to the guide strand [37]. We demonstrated for the first time that miR‐21‐3p is increased by muscle damage and negatively correlates with *Ppargc1a* expression, but whether miR‐21‐5p has similar effects warrants further research.

Previous studies showed that miRNAs 23, 26, 27 and 29 participate in muscle–kidney crosstalk, affecting kidney function [38, 39, 40]. However, these studies mainly show that boosting miRNA levels can improve renal function via muscle–kidney crosstalk, but do not demonstrate that reduced miRNA levels contribute to renal dysfunction in CKD. In contrast, our data showed that an increase in miR‐21 in muscle directly contributes to renal fibrosis development, providing a potential target for kidney disease with muscle wasting. Recent studies have shown that anti‐miR‐21 oligonucleotides are effective and specific in animal models of diabetic kidney and Alport syndrome [41, 42]. These findings support their potential as novel therapeutics, exemplified by lademirsen, a miR‐21 inhibitor currently in clinical trials for Alport syndrome (NCT02855268).

A key strength of our study is the use of experimental models designed to investigate damaged muscle as an initial culprit in driving kidney injury. However, our model could differ from conditions in patients with CKD, such as uremia‐induced muscle injury. Although we compared miRNAs altered in fibrotic kidneys with those in denervated muscle, it remains uncertain whether EVs from patients with CKD and muscle atrophy carry similar cargo. Another limitation is the exclusive use of male mice. Since sex‐specific regulation of metabolism and neuromuscular signalling may influence the response to muscle denervation and the trajectory of kidney disease [43, 44] further studies including female mice are warranted to determine whether similar mechanisms operate across sexes and to identify any potential sex‐specific responses. To note, there is age discrepancy between our murine models (young adult mice) and the human data (older adults), which may limit the direct translational interpretation of our findings. Given that muscle wasting and CKD is age‐related phenomena, the observations in older human subjects enhance the clinical relevance of the study. Nonetheless, whether the effect of muscle denervation on kidney injury observed in our mouse model is reproduced in aged animal models remains to be investigated.

In conclusion, our findings provide novel evidence that skeletal muscle injury can serve as an upstream contributor to kidney disease, offering new insights into the muscle‐kidney axis and its potential to form a vicious cycle. These results also highlight the therapeutic potential of targeting small EV‐mediated miRNA delivery for the treatment and prevention of renal disease.

## Conflicts of Interest

The authors declare no conflicts of interest.

## Supporting information


**Table S1.** The Characteristics of Study Subjects.
**Table S2.** Primer sequences used for qPCR analysis.
**Table S3.** Primary antibodies used in the present study.
**Table S4.** Commonly downregulated genes in denervated muscle and fibrotic kidney.
**Table S5.** Commonly upregulated miRNAs in denervated muscle and fibrotic kidney.
**Figure S1.** Reduced muscle mass aggravates adenine‐diet‐induced kidney injury.
**Figure S2.** Long‐term muscle denervation leads to kidney injury.
**Figure S3.** The gene expression of small EV production and release markers in denervated muscle, TGFβ‐, and dexamethasone‐treated C2C12 cells.
**Figure S4.** The expression of EVs markers in kidney of UUO mice.
**Figure S5.** Inhibition of muscle EV synthesis/secretion by intramuscular GW4869 injection ameliorates kidney injury in denervated UUO mice.
**Figure S6.** Gene ontology enrichment analysis of commonly downregulated genes in denervated muscle and fibrotic kidney.
**Figure S7.** miR‐21a inhibitor ameliorates TGFβ1‐induced mProx24 cell injury.

## Data Availability

This study does not include any data that need to be deposited in external repositories. Data supporting the findings of this study are available upon request from the corresponding authors.
