# Supplementary material for: Small Extracellular Vesicles Derived From Damaged Muscle Aggravate Kidney Injury Progression
Source: J Cachexia Sarcopenia Muscle. 2025 Jun 13;16(3):e13861. doi: 10.1002/jcsm.13861 (PMC12163521; doi:10.1002/jcsm.13861)
Supplement: Supplementary file 1 — Table S1. The Characteristics of Study Subjects. Table S2. Primer sequences used for qPCR analysis. Table S3. Primary antibodies used in the present study. Table S4. Commonly downregulated genes in denervated muscle and fibrotic kidney. Table S5. Commonly upregulated miRNAs in denervated muscle and fibrotic kidney. Figure S1. Reduced muscle mass aggravates adenine‐diet‐induced kidney injury. Figure S2. Long‐term muscle denervation leads to kidney injury. Figure S3. The gene expression of small EV production and release markers in denervated muscle, TGFβ‐, and dexamethasone‐treated C2C12 cells. Figure S4. The expression of EVs markers in kidney of UUO mice. Figure S5. Inhibition of muscle EV synthesis/secretion by intramuscular GW4869 injection ameliorates kidney injury in denervated UUO mice. Figure S6. Gene ontology enrichment analysis of commonly downregulated genes in denervated muscle and fibrotic kidney. Figure S7. miR‐21a inhibitor ameliorates TGFβ1‐induced mProx24 cell injury. [file JCSM-16-e13861-s001.docx]

**SUPPLEMENTARY MATERIAL**

**Small extracellular vesicles derived from damaged muscle aggravate the progression of kidney injury**

Songling Jiang, Seunghee Kang, Oran Kwon, Wonhyo Seo, Eun-Jung Jin, Hunjoo Ha, Joo Young Huh

**Supplementary Tables**

**Table S1.** The Characteristics of Study Subjects

**Table S2.** Primer sequences used for qPCR analysis

**Table S3.** Primary antibodies used in the present study

**Table S4.** Commonly downregulated genes in denervated muscle and fibrotic kidney

**Table S5.** Commonly upregulated miRNAs in denervated muscle and fibrotic kidney

**Supplementary Figures**

**Figure S1.** Reduced muscle mass aggravates adenine-diet-induced kidney injury

**Figure S2.** Long-term muscle denervation leads to kidney injury

**Figure S3.** The gene expression of small EV production and release markers in denervated muscle, TGFβ-, and dexamethasone-treated C2C12 cells

**Figure S4.** The expression of EVs markers in kidney of UUO mice

**Figure S5.** Inhibition of muscle EV synthesis/secretion by intramuscular GW4869 injection ameliorates kidney injury in denervated UUO mice

**Figure S6.** Gene ontology enrichment analysis of commonly downregulated genes in denervated muscle and fibrotic kidney

**Figure S7.** miR-21a inhibitor ameliorates TGFβ1-induced mProx24 cell injury

**Supplementary Tables**

**Table S1. The characteristics of study subjects**

| **Variables** | **Control** | **Exercise + KME** |
| --- | --- | --- |
| Gender (M/F) | 1/8 | 2/7 |
| Age (y) | 65.6±1.5 | 62.2±2.3 |
| BMI (kg/m^2^) | 25.2±0.8 | 23.4±1.0 |
| Body weight (kg) | 60.4±2.3 | 59.3±3.0 |
| Skeletal muscle mass (kg) | 39.2±1.6 | 46.0±2.8 ***** |
| Fat free mass (kg) | 41.6±1.7 | 49.0±3.1 ***** |
| Soft lean mass (kg) | 22.3±1.0 | 27.7±2.3 ***** |
| Fat mass (kg) | 18.9±1.0 | 10.4±1.0 ***** |
| Percent Body fat (%) | 31.2±1.3 | 17.9±1.7 ***** |

The exercise group performed knee-strengthening resistance exercises (1 set at 10RM, 3 times/week) and received Korean mistletoe extract (2 g/day) for 12 weeks. The control group received no intervention. Statistical significance was determined using an unpaired t-test. * P < 0.05

**Table S2.** **Primer sequences used for qPCR analysis**

| **Gene** | **Primer sequences** | |  |
| --- | --- | --- | --- |
| *18s* | F: CGAAAGCATTTGCCAAGAAT | R: AGTCGGCATCGTTTATGGTC | |
| *Atrogin-1* | F: AGACCGGCTACTGTGGAAGAG | R: CCGTGCATGGATGGTCAGTG | |
| *Cd9* | F: TTCTGTCCCAGTCGTTCGTG | R: CTGAGAGTCGAATCGGAGCC | |
| *Cd81* | F: GATAGTGACTCTCGCGCCTC | R: GCTACACCTAGGATCACGCC | |
| *Col1* | F: GAACATCACCTACCACTGCA | R: GTTGGGATGGAGGGAGTTTA | |
| *Col4* | F: AACAACGTCTGCAACTTCGC | R: CTTCACAAACCGCACACCTG | |
| *Cox4i2* | F: GGGCAGCTCTGGATAGTTCC | R: CTCATTACCAGACTCCGGGC |  |
| *Cytb* | F: AAGAGCACCTGGGTGATCCTGCA | R: CGTGCATCCGTAGAGTGCCCG |  |
| *Cytc* | F: GAACGTTCGTGGTGTTGACC | R: CCGTGGAGATTTGGTCCAGT |  |
| *F4/80* | F: CTGTAACCGGATGGCAAACT | R: ATGGCCAAGGCAAGACATAC |  |
| *Il6* | F: AGTTGCCTTCTTGGGACTGA | R: TCCACGATTTCCCAGAGAAC |  |
| *Mcp1* | F: CTTCTGGGCCTGCTGTTCA | R: CCAGCCTACTCATTGGGATCA |  |
| *Mcad* | F: CAACACTCGAAAGCGGCTCA | R: ACTTGCGGGCAGTTGCTTG |  |
| *mtDNA* | F: CCACTTCATCTTACCATTTA | R: ATCTGCATCTGAGTTTAATC |  |
| *Myog* | F: CAGTTGGGCATGGTTTCGTC | R: AATGCACTGGAGTTCGGTCC |  |
| *Ppargc1a* | F: TCGATGTGTCGCCTTCTTGC | R: ACGAGAGCGCATCCTTTGG |  |
| *Ppara* | F: GAGAGGGCACACGCTAGGAA | R: GAACACCAATGTTCGGAGCC |  |
| *Rab11b* | F: CTTGCACTGTTGCCCGTTAC | R: GTCTGCCCACTGTCTTCAGC |  |
| *Rab35* | F: GAAACGCTGCTGCTAATGCC | R: AATCAGTCCACTCAGCTGGC |  |
| *Acta2* | S: GTCCCAGACATCAGGGAGTAA | AS: TCGGATACTTCAGCGTCAGGA |  |
| *Tgfb1* | F: CTTTAGGAAGGACCTGGGTT | R: CAGGAGCGCACAATCATGTT |  |
| *Vcam1* | F: ACAGACAGTCCCCTCAATGG | R: ACCTCCACCTGGGTTCTCTT |  |
| miR-21a-3p | F: CAACAGCAGUCGAUGGGCUGUC |  |  |

**Table S3. Primary antibodies used in the present study**

| Antibody | Catalog number | Company |
| --- | --- | --- |
| anti-Alix | Sc-53540 | Santa Cruz Biotechnology |
| anti-CD9 | EXOAB-CD9A-1 | System Biosciences |
| anti-CD63 | ab217345 | Abcam |
| anti-CD81 | #10037 | Cell Signaling Technology |
| anti-COL1 | 1310-01 | Southern Biotech |
| anti-FN | sc-6952 | Santa Cruz Biotechnology |
| anti-GAPDH | sc-32233 | Santa Cruz Biotechnology |
| anti-αSMA | ab5694 | Abcam |
| anti-TSG101 | ab125011 | Abcam |

Abcam, Cambridge, MA, USA; Cell Signaling Technology, Danvers, MA, USA; Developmental studies hybridoma bank (DSHB), Iowa, USA; Santa Cruz Biotechnology, Inc., Dallas, TX, USA; Southern Biotech, Birmingham, AL, USA; System Biosciences, Palo Alto, CA, USA

**Table S4.** **Commonly downregulated genes in denervated muscle and fibrotic kidney**

| Ulk2 | Cluh | Retsat | Glrx2 | Uqcr10 |
| --- | --- | --- | --- | --- |
| Mrpl35 | Cpt2 | Aldh1a1 | Isca1 | Acsl1 |
| Slc11a2 | Atp5d | Cyc1 | Aktip | Fam69a |
| Mlf2 | Kmt5a | Got1 | Arl8b | Dnajc15 |
| Ggact | Fam220a | Ldha | Ndufa8 | Aqp7 |
| Ndufa1 | Ndufb2 | Tigar | Mrpl42 | Ndufa5 |
| Dirc2 | Rida | Gpt2 | Sdhc | Lims2 |
| Mrps23 | Ghr | Idh3a | 1700021F05Rik | Spsb4 |
| Cox4i1 | Mtfp1 | Ndufaf5 | Abhd5 | Ndufb8 |
| Pdha1 | Krt10 | Fn3k | Mkks | Suclg1 |
| Got2 | Ndufc1 | Ky | Tmem126a | Tfrc |
| Tinag | Tmem94 | Mgst3 | Adk | Poldip2 |
| Tspan7 | Mrpl34 | Ndufb3 | Cox10 | Fech |
| Dnajc19 | Tusc2 | Ndufa12 | Llgl2 | Mfn1 |
| Pcmt1 | Nudt8 | Herc3 | Alas1 | Msx1 |
| Nqo1 | Fam19a4 | Rhot2 | Prdx5 | Slc25a19 |
| Acadvl | Ndufa4 | Msrb2 | Kcng4 | Cox6b1 |
| Mapk14 | Eci1 | Tarsl2 | Cs | Slc38a3 |
| Ndufa3 | Arpc5l | Atp5f1 | Strbp | Pex11a |
| Ndufaf1 | Tmem242 | Mrpl18 | Atp5g1 | Mrpl47 |
| Adsl | Mmab | Prkaa2 | Tpi1 | Ppargc1a |
| Cisd1 | Abhd11 | Cyhr1 | Ptpmt1 | Cox7a2 |
| Ppif | Chchd10 | Opa3 | Paqr9 | Ddo |
| Fuom | Vegfb | Pccb | Sars | Pla2g12a |
| Magix | Tdrp | Mrpl51 | Selenbp1 | Ethe1 |
| Coq7 | Slc25a12 | Hdlbp | Mfn2 | Ech1 |
| Ndufs6 | Maf | Ndufb9 | Pnkd | Glrx5 |
| Hras | Dlat | Ecsit | Stradb | Ndufb6 |
| Gys1 | Acot13 | Slc25a3 | Alpl | Mavs |
| Nqo2 | Syngr1 | Rab4a | Mrpl12 | Akap1 |
| Higd2a | Cox5a |  |  |  |

**Table S5. Commonly upregulated miRNAs in denervated muscle and fibrotic kidney**

| miR-1949 | miR-27a | miR-21a-5p |
| --- | --- | --- |
| miR-142a-5p | miR-34b-5p | miR-674-3p |
| miR-34c-5p | miR-142a-3p | miR-21a-3p |

**Supplementary Figures**

**
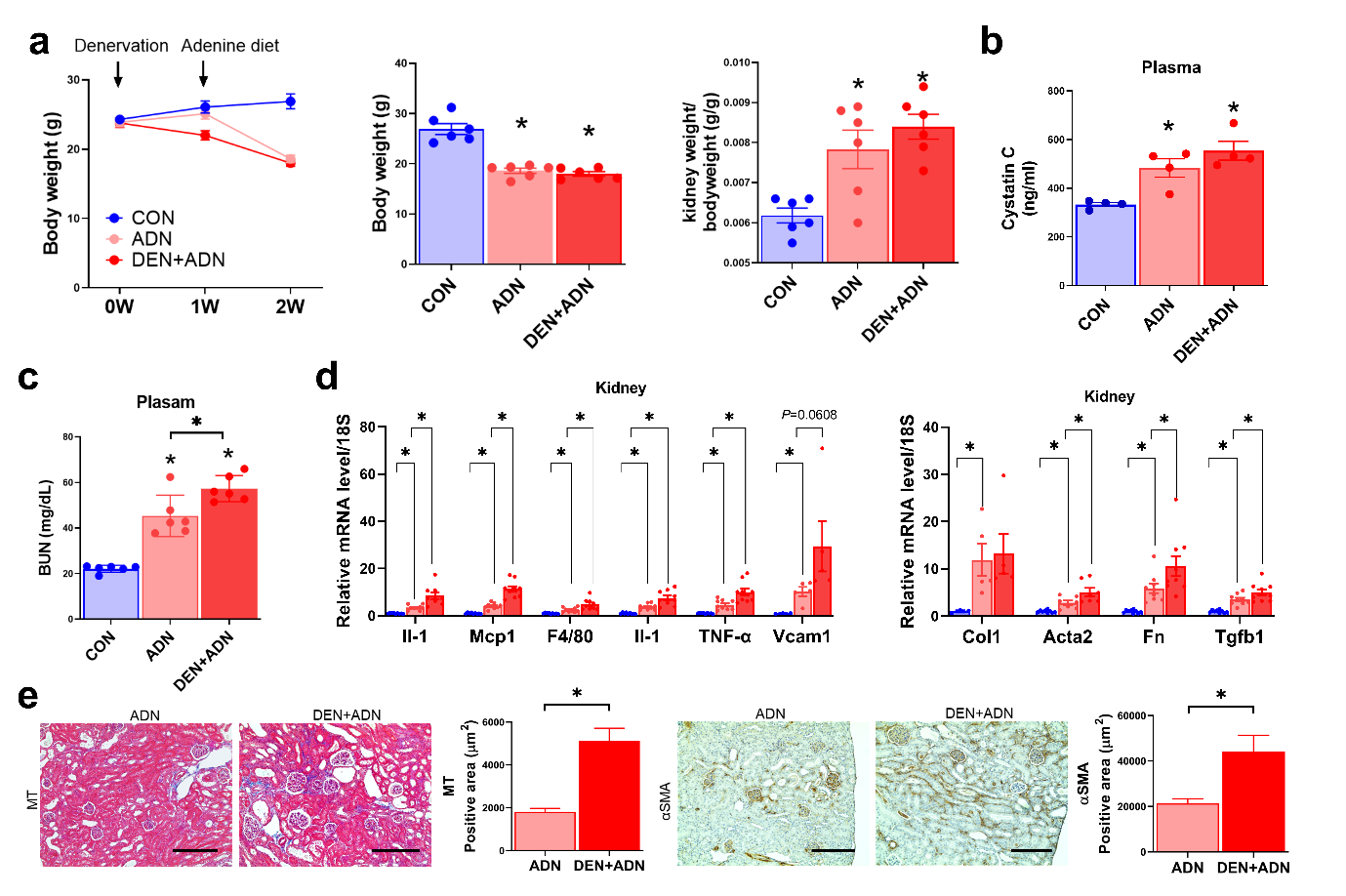
** **Figure S1.** Reduced muscle mass aggravates adenine-diet-induced kidney injury. (a) Body weight and kidney weight to body weight ratio were measured. (b and c) Plasma cystatin C and BUN levels were measured by ELISA. (d) The expression of genes related to kidney inflammation and fibrosis were measured by qPCR. (e) Kidney sections were stained with Masson's trichrome and anti-αSMA antibody, and the area of positive staining were quantified using Image-Pro Plus. Original magnification, 100×; scale bar, 200 μm. Data are expressed as the mean ± standard error of five mice/group. Statistical significance was determined using one-way ANOVA followed by Tukey’s post hoc test for multiple comparisons. *p < 0.05. CON, control; ADE, adenine-diet fed mice; DEN, denervation: BUN, blood urea nitrogen; MT, Masson’s trichrome.

**
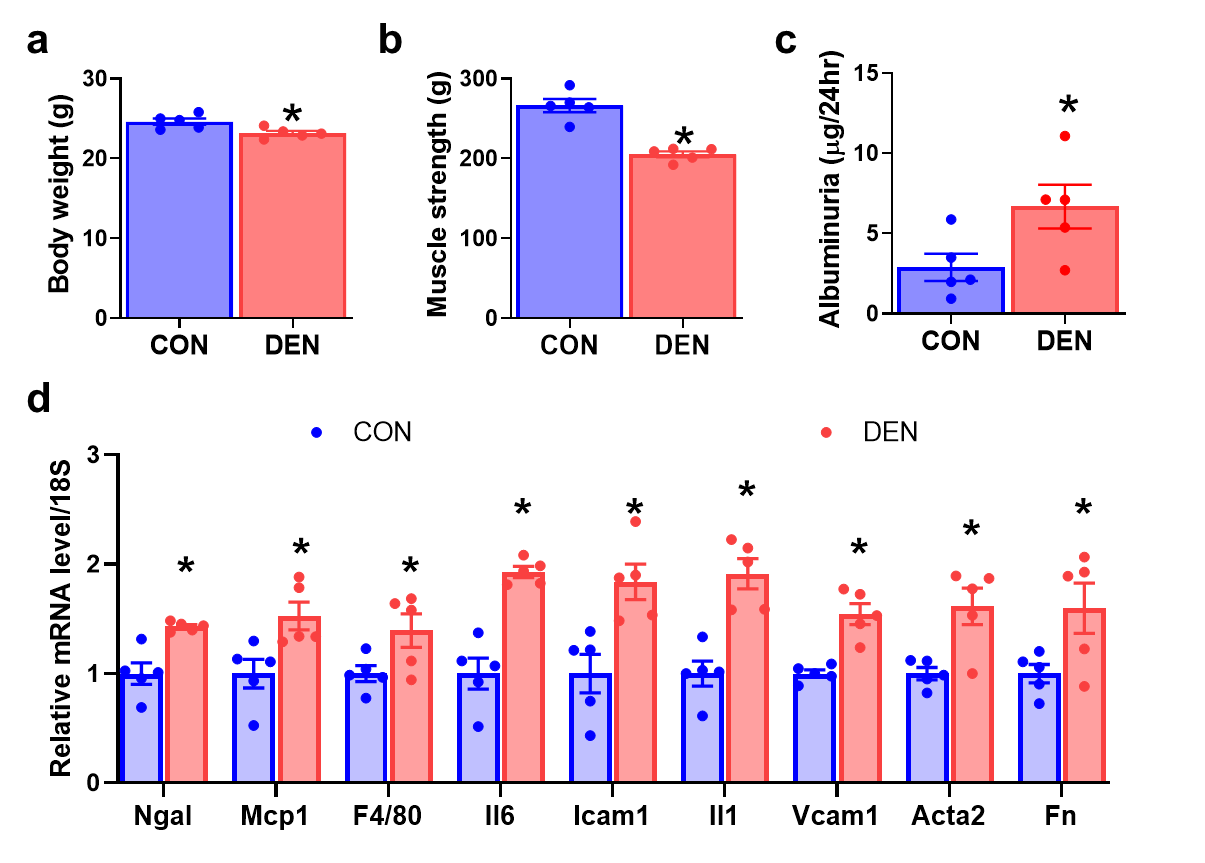
Figure S2.** Long-term muscle denervation leads to kidney injury. 6-week-old male mice were subjected to unilateral tibial nerve denervation and sacrificed after 4 weeks. (a–b) Body weight and muscle strength were measured. (c) Urinary albumin excretion was detected by ELISA. (d) The expressions of genes related to kidney inflammation and kidney fibrosis were measured by qPCR. Data is expressed as the mean ± standard error of five mice/group. Statistical significance was determined using one-way ANOVA followed by Tukey’s post hoc test for multiple comparisons. *p < 0.05 vs. CON mice. CON, control; DEN, denervation.


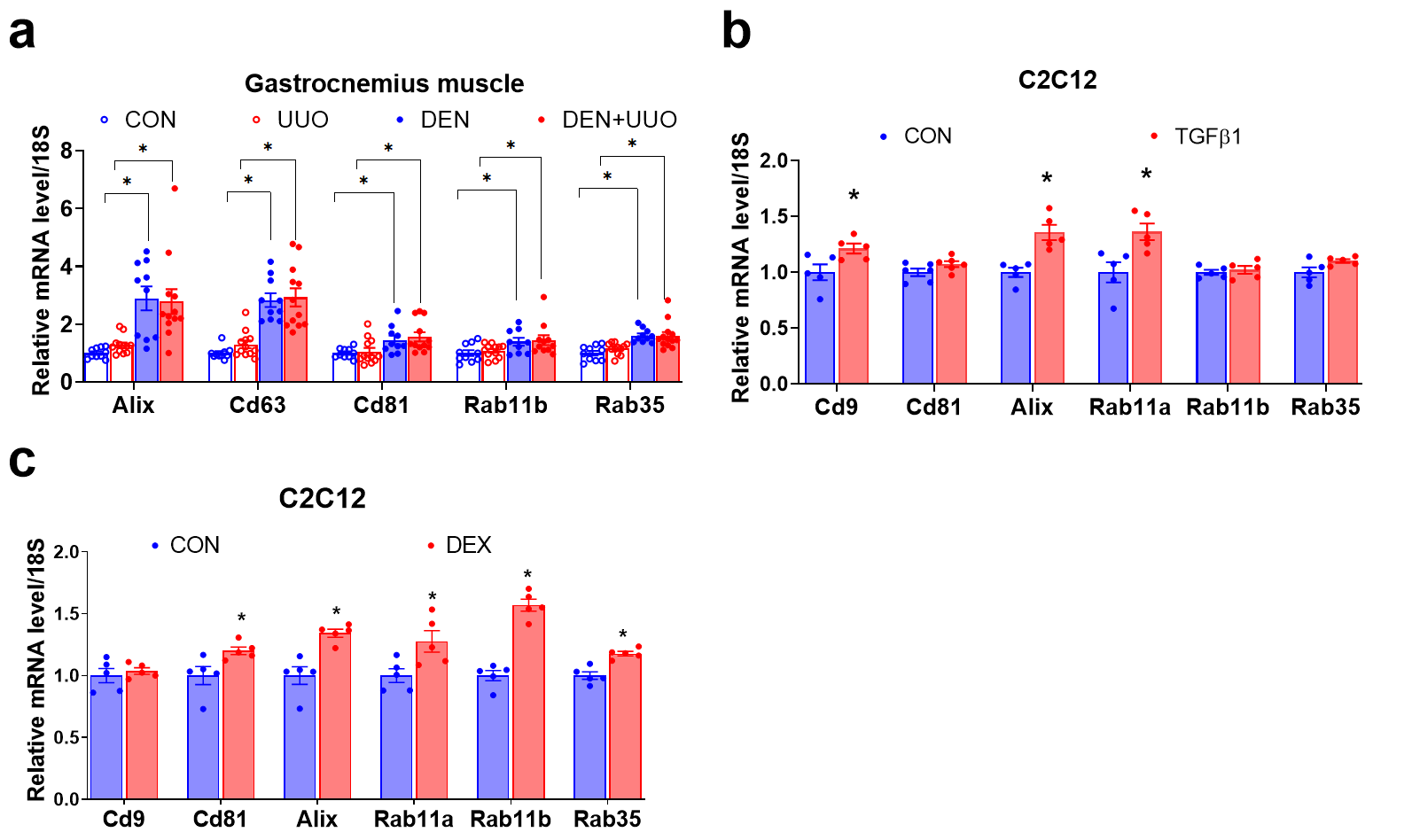


**Figure S3.** The gene expression of small EV production and release markers in denervated muscle, TGFβ-, and dexamethasone-treated C2C12 cells. (a-c) Small EV markers (*Cd9*, *Cd81*, and *Alix*) and EV release-related genes (*Rab11a, Rab11b,* and *Rab35*) in (a) gastrocnemius muscle, (b) TGFβ1 (10 ng/ml, 8 h), and (c) dexamethasone (100 μM, 24 h) treated C2C12 cells using qPCR (n = 4). Data are expressed as the mean ± standard error. Statistical significance was determined using one-way ANOVA followed by Tukey’s post hoc test for multiple comparisons. *p < 0.05 vs. control. CON (Control); UUO (Unilateral ureteral obstruction only); DEN (Muscle denervation only); DEN+UUO (UUO surgery performed after muscle denervation); EVs, extracellular vesicles; DEX, dexamethasone.

**
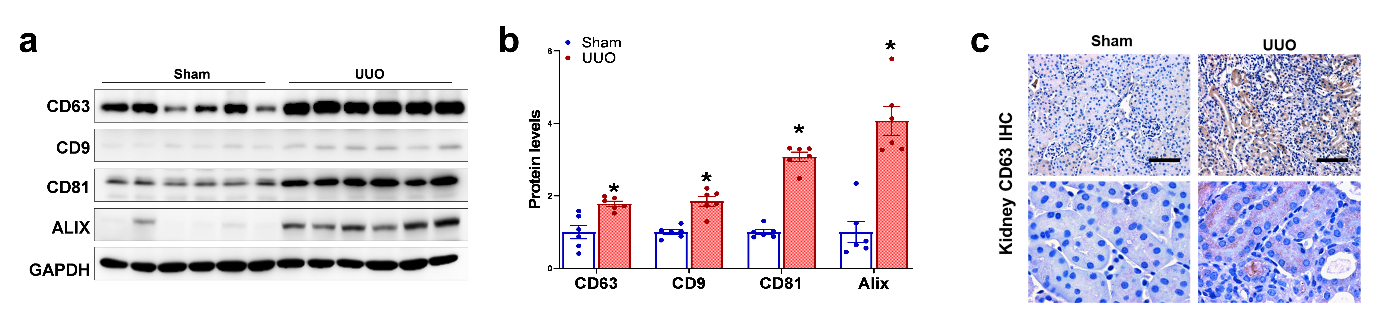
** **Figure S4.** The expression of EVs markers in kidney of UUO mice. (a-c) The protein levels of EVs markers in sham and obstructed kidney were measured by western blot and anti-CD63 immunohistochemical (IHC) staining. Original magnification, 100×; scale bar, 200 μm. Data are expressed as the mean ± standard error of 5 mice/group. Statistical significance was determined using one-way ANOVA followed by Tukey’s post hoc test for multiple comparisons. *p < 0.05 vs. sham mice. EVs, extracellular vesicles; UUO, unilateral ureteral obstruction


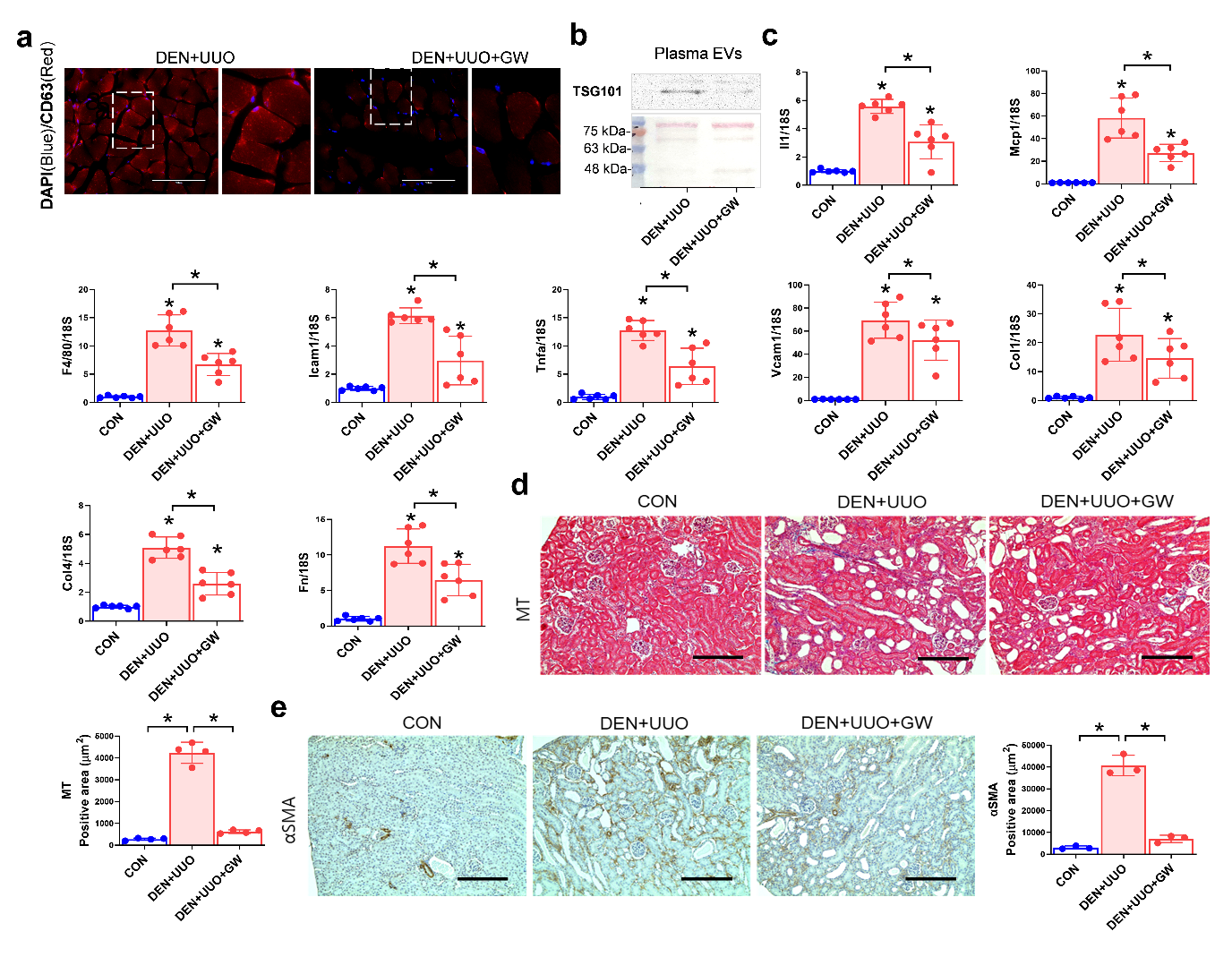
 **Figure S5.** Inhibition of muscle EV synthesis/secretion by intramuscular GW4869 injection ameliorates kidney injury in denervated UUO mice. (a) Representative images from immunofluorescence staining of CD63 in gastrocnemius muscle in denervated UUO mice with and without GW4869 treatment. (b) The amount of plasma EVs were measured by TSG101 protein expression. (c) The expressions of genes related to kidney inflammation and fibrosis were measured by qPCR. (d and e) Kidney sections were stained with Masson's trichrome and anti-αSMA antibody, and the area of positive staining were quantified using Image-Pro Plus. Original magnification, 100×; scale bar, 200 μm. Data are expressed as the mean ± standard error of six mice/group. Statistical significance was determined using one-way ANOVA followed by Tukey’s post hoc test for multiple comparisons. *p < 0.05. EVs, extracellular vesicles; UUO, unilateral ureteral obstruction; DEN, denervation; αSMA, alpha smooth muscle actin; COL1, Collagen I.


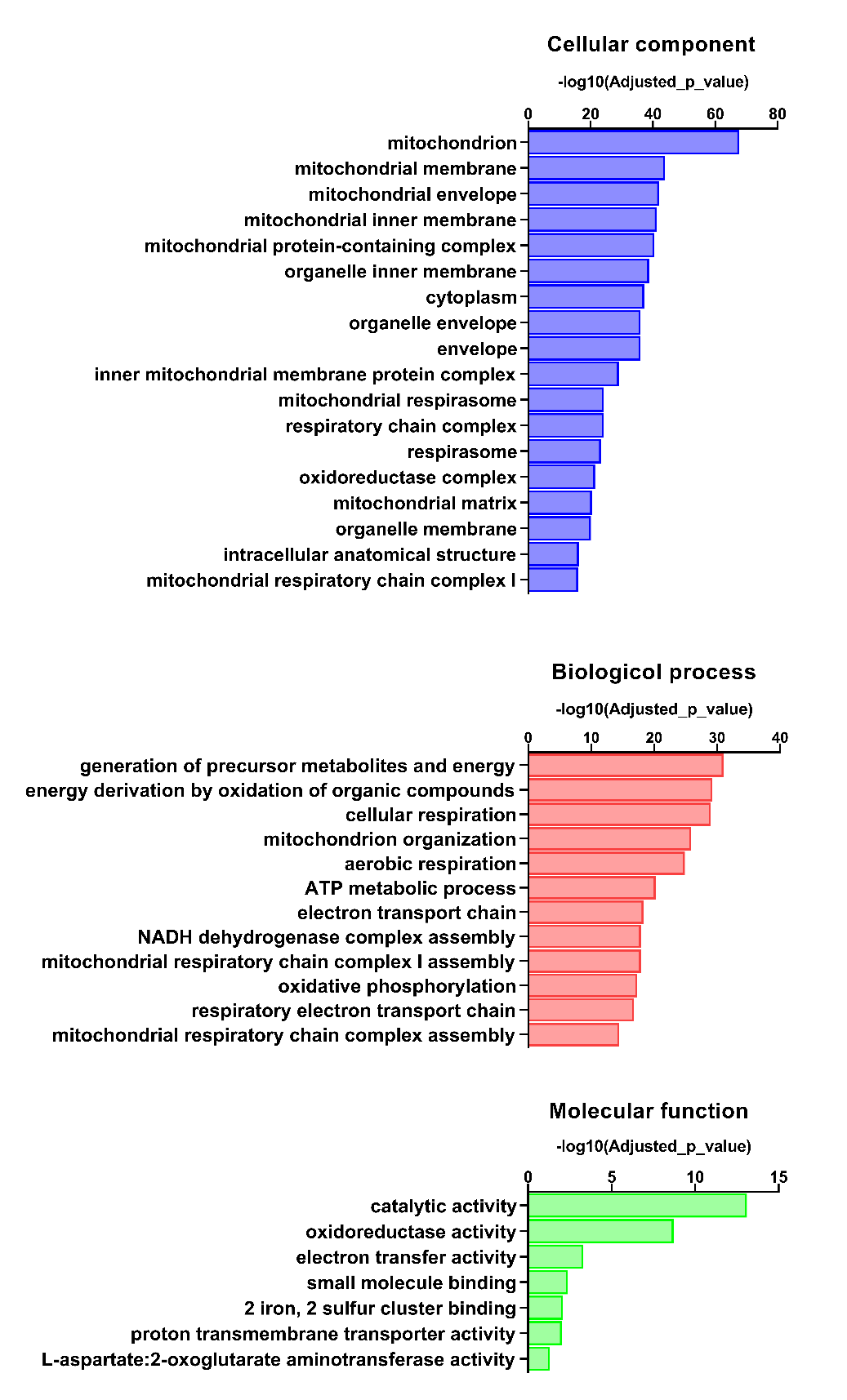


**Figure S6.** Gene ontology enrichment analysis of commonly downregulated genes in denervated muscle and fibrotic kidney.


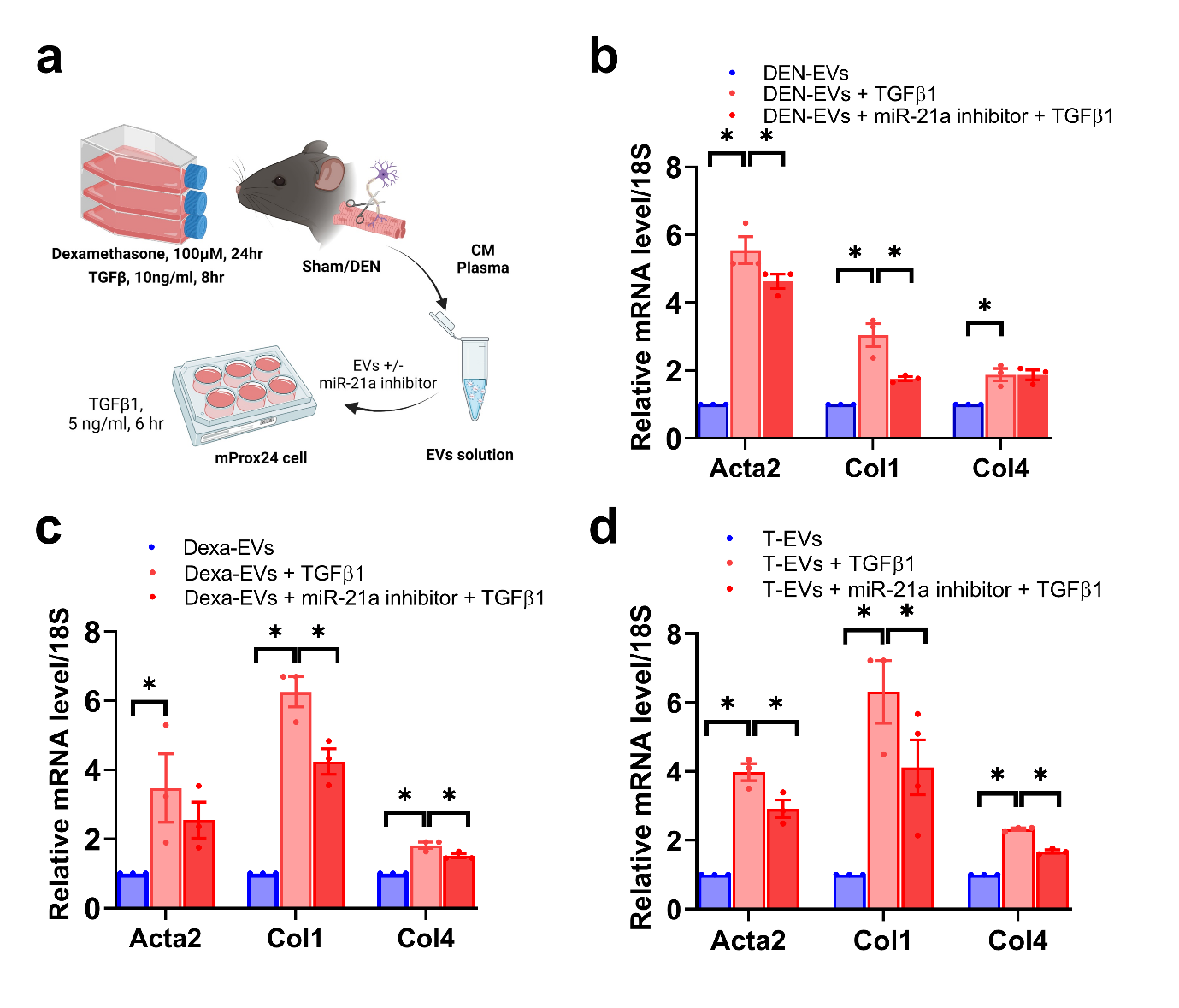
 **Figure S7.** miR-21a inhibitor ameliorates TGFβ1-induced mProx24 cell injury. (a) In vitro experimental design. EVs were isolated from plasma of denervated mice or conditioned medium of dexamethasone- or TGFβ1-treated C2C12cells. mProx24 cells were pretreated with isolated EVs and miR-21a inhibitor and then stimulated with TGFβ1 (5 ng/mL, 6 h). mProx24 cells were pretreated with EVs derived from (b) plasma of denervated mice, (c) conditioned media of dexamethasone treated C2C12 cells, and (d) conditioned media of TGFβ1 treated C2C12 cells. Data are expressed as the mean ± standard error of three sets. Statistical significance was determined using one-way ANOVA followed by Tukey’s post hoc test for multiple comparisons. *p <0.05. DEN, denervation; Dexa, dexamethasone; T, TGFβ1; EV extracellular vesicles.
